# Identification and Expression of Nine Oak Aquaporin Genes in the Primary Root Axis of Two Oak Species, *Quercus petraea* and *Quercus robur*

**DOI:** 10.1371/journal.pone.0051838

**Published:** 2012-12-17

**Authors:** Claire Rasheed-Depardieu, Claire Parent, Michèle Crèvecoeur, Julien Parelle, Fabienne Tatin-Froux, Grégoire Le Provost, Nicolas Capelli

**Affiliations:** 1 Université de Franche-Comté, UMR 6249 Chrono-Environnement, Besançon, France; 2 Département de Botanique et Biologie végétale, Université de Genève, Genève, Suisse; 3 UMR 1202 BIOGECO, INRA, Cestas, France; 4 Université de Bordeaux, UMR 1202 BIOGECO, Talence, France; University of Nottingham, United Kingdom

## Abstract

Aquaporins (AQPs) belong to the Major Intrinsic Protein family that conducts water and other small solutes across biological membranes. This study aimed to identify and characterize AQP genes in the primary root axis of two oak species, *Quercus petraea* and *Quercus robur*. Nine putative AQP genes were cloned, and their expression was profiled in different developmental root zones by real-time PCR. A detailed examination of the predicted amino acid sequences and subsequent phylogenetic analysis showed that the isolated AQPs could be divided into two subfamilies, which included six plasma membrane intrinsic proteins (PIPs) and three tonoplast intrinsic proteins (TIPs). We characterized the anatomical features of the roots and defined three developmental root zones: the immature, transition and mature zones. Expression analysis of the AQPs was performed according to these root developmental stages. Our results showed that the expression of *PIP2;3* and *TIP1* was significantly higher in *Quercus petraea* compared with *Quercus robur* in the three root zones. However, *PIP2;1* and *TIP2;1* were found to be differentially expressed in the mature zone of the two oak species. Of the nine AQP genes identified and analyzed, we highlighted four genes that might facilitate a deeper understanding of how these two closely related tree species adapted to different environments.

## Introduction

The maintenance of an optimal water balance is crucial for plant survival. In the soil-plant-atmosphere-continuum, water is transported radially across the root tissues and axially to the aerial part of the plant. Radial tissues impose a major resistance to water movement in roots that can occur through the apoplastic and cell-to-cell pathways [1]. The apoplastic pathway allows water transport via intercellular spaces and across cell walls, and the relative contribution of this pathway to the global water transport within the root varies with the developmental stages of the root. In differentiated endodermal and hypodermal tissues, the presence in the root cell walls of a Casparian strip, which is composed of the hydrophobic substance suberin, severely restricts water transport through the apoplastic way [2], and water molecules are forced to transit cellular membranes via water channels called aquaporins (AQPs) [3]. AQPs belong to a large family of highly conserved proteins, called Major Intrinsic Proteins (MIPs), which include PIPs (plasma membrane intrinsic proteins), TIPs (tonoplast intrinsic proteins), NIPs (nodulin 26-like intrinsic proteins), SIPs (small intrinsic proteins) and XIPs (X intrinsic proteins) [4]. These proteins are known to transport water molecules and small solutes through biological membranes. In plants, MIPs are particularly abundant and have multiple isoforms [5]. AQPs have been identified in different herbaceous model plants, such as *Arabidopsis thaliana*, *Oryza sativa* and *Gossypium hirsutum*, based on whole genome analysis [6], [7], [8]. In woody species, 56 MIPs were identified in *Populus trichocarpa* and 28 in *Vitis vinifera* [9], [10], but little is known about AQPs in other common tree species, such as walnut, olea, beech and oaks.

Sessile (*Quercus petraea* (Matt.) Liebl.) and pedunculate (*Quercus robur* L.) oaks are two forest tree species that predominate the northern hemisphere. These two species are closely related [11] at the genetic level, but they exhibit different ecological exigencies. *Quercus robur* naturally occur in hydromorphic soils in which water-logging is frequent, whereas *Quercus petraea* is restricted to deep, acidic and well-drained soils [12]. The natural repartition of these two oaks species could be attributed to differences in their hydraulic properties. In four years-old trees, Nardini et al. (1999) previously shown that the root hydraulic conductivity in drought tolerant species *Quercus suber*, *Quercus pubescens* and *Quercus petraea* was lower compared to drought sensible species, namely *Quercus alba*, *Quercus cerris*, *Quercus robur* and *Quercus rubra* [13]. Young *Quercus robur* seedlings also exhibited a significantly higher root hydraulic conductivity than *Quercus petraea* [14]. Modulation in root hydraulic properties were previously shown to be influenced by the activity of AQPs [15], [16], [17]. However, the functional link between the expression of AQPs and water transport at whole plant level remains unclear. In some cases, transgenic approaches have demonstrated the role of individual isoforms in root water transport [18], [19], whereas other studies have suggested that some AQP members act redundantly to facilitate water transport in plants [20], [21]. Thus, AQPs seem to play an important role in the regulation of the water balance in plants and facilitate tree adaption to stressful environmental conditions [22]. The aim of this work was to identify and characterize oak genes encoding AQPs potentially important for the regulation of root water flow. Under standard conditions, the comparative expression analysis of the identified genes uncovered potential regulatory pathways: these findings might facilitate understanding of how these two sympatric species adapted to their specific environment during the course of evolution. In this regard, we first measured root hydraulic conductivity in root systems of *Quercus petraea* and *Quercus robur*. Then, root anatomy was examined at different distances from the root tip to look for the presence of suberin deposits, and AQP expression was measured in different developmental zones along the primary root.

## Materials and Methods

### Plant Material and Growth Conditions

*Quercus petraea* and *Quercus robur* acorns harvested in north-eastern France were provided by the Office National des Forêts (ONF, 153 avenue Edouard Herriot, 39300 Champagnole, France/Phone: +333 84 52 53 95), which is an authorized and recommended agency that supplies the laboratory with cataloged plant material, and stored at 4°C until use. No specific permits were required for the described field studies. We can confirm that *Quercus petraea* and *Quercus robur* are not included in the list of endangered or protected species. Acorns were shelled and left to germinate in vermiculite for one week. Individual acorns were grown in a 1.8-L pot containing river sand for four weeks in a growth chamber under controlled environmental conditions as previously described [23]. The experimental design consisted of three experimental blocks arranged in three separate containers. Each block represented 7 individuals of *Quercus petraea* and *Quercus robur* that were completely randomized in each container. Each seedling was individually irrigated twice a day using a commercial fertilizer solution (0.8 mL per L, NPK 6/6/6, SEM, Germany) in an automated *Ebb-and-Flow* system.

### Root Pressure Probe Measurements

The hydraulic conductance of root systems (L*r*) was measured using a root pressure probe (Bayreuth University, Germany), according to Steudle and Meshcheryakov [24]. Root surface aera (A*r*) was determined using WinRHIZO® (Regent Instruments, Montreal, QC, Canada), assuming that in river sand the overall root system corresponds to the active water absorption zone. Root hydraulic conductivity (Lp*r*) was calculated by dividing the root hydraulic conductance by the root surface area.

### Sample Collection Procedure

After 4 weeks, which corresponded with the first mature leaf flush, the root systems were gently washed in water, and the main root apex (the last 4 cm of the root tip) was excised using a razor blade and processed. Three segments were excised at different distances from the root tips: first (0–1 cm), second (1–2 cm) and third (2–4 cm). The segments were immediately frozen in liquid nitrogen and stored at −80°C until RNA extraction was performed. During each experiment, the root samples were collected at seven different time points during the day to minimize potential transcriptional variation due to diurnal effects. To obtain sufficient plant material for RNA extraction, the collected plant material was pooled from three independent plants representing each block for each time point. The complete experiment was repeated three times to test the reproducibility of the results.

### Detection of Apoplastic Barriers

Fresh 100-µm thick cross sections were cut at 1, 2, 3 and 4 cm from the primary root tip of both oak species using a vibratome. The sections were immediately stained with 0.1% berberine hemisulfate (Sigma Chemical, St Louis, U.S.A.) for 20 min and observed under UV illumination (excitation 377/50 nm; emission 542/27 nm) using a Nikon eclipse 80i microscope (Nikon, Japan) to detect suberin (bright blue signal). Longitudinal sections through the first centimeter of root tips fixed with FAA (3.7% formaldehyde, 60% ethanol, and 5% acetic acid) and embedded in paraffin, were stained with hematoxylin for 20 min (Merck, Damstadt, Germany). These sections were used to measure the length of cortical cells at different distances from the root cap junction. All of the sections were photographed with a Nikon Digital Color Camera Sight DS-Fi-1 (Nikon, Japan).

### Cloning of Putative AQP cDNA Sequences in *Quercus petraea* and Phylogenetic Analysis

Partial cDNAs encoding nine potential AQPs were preliminarily identified from SSH libraries prepared from 4-cm oak root tips (Table S1, Figure S1) [25]. Total RNA of 4-cm oak root tip was extracted from root material of *Quercus petraea*. Completed cDNAs were obtained by performing RACE-PCR (SMARTer*™* RACE cDNA Amplification kit, Clontech, Mountain View, U.S.A.) according to the manufacturer’s instructions. The resulting PCR products were purified using the MinElute® Gel Extraction kit (Qiagen, Hilden, Germany), ligated into the pGEM®-T Easy vector (Promega, Madison, U.S.A.) and cloned into the *Escherichia coli* JM109 strain. Because high homology was found within the coding region, specific primers with divergent 3′ and 5′ untranslated regions were used to amplify the complete coding DNA sequence. The selected clones and PCR products were sequenced (Millegen, Labège, France). Details regarding the PCR conditions and a list of primers used for RACE-PCR and the amplification of the full-length coding regions are provided in Table S2. The AQP topology was determined using TMpred software (http://www.ch.embnet.org/software/TMPRED_form.html) and the OCTOPUS program (http://octopus.cbr.su.se/) [26], [27] with default parameters. For phylogenetic analysis, the amino acid sequences from *Quercus petraea* and representative plants were aligned using the MUSCLE program in MEGA 5 software (http://www.megasoftware.net/) [28]. The accession numbers of the sequences used to construct the phylogenetic tree are detailed in Table S3. The resulting alignments were inspected and realigned manually if necessary. The phylogenetic tree was constructed using the maximum likelihood method according to the JTT Model with 1,000 bootstrap replicates.

### Selection and Analysis of Housekeeping Genes

Six candidates commonly used for normalization in real-time PCR applications in other plant species were selected: elongation factor 1 alpha, cyclophylin, polyubiquitin, alpha-tubulin, membrane H^+^ ATPase and actin. To design specific primers, the corresponding sequences were identified from the available *Quercus petraea* and *Quercus robur* sequences in GenBank and the first oak unigene set generated by Uneo et al. (2010) [29]. The collected sequences were aligned using the ClustalW program (http://www.ebi.ac.uk/Tools/msa/clustalw2/) [30] (see Table S4 for details).

### Expression Analysis of Oak AQP Genes

Total RNA was extracted (RNeasy® Plant Mini kit, Qiagen, Hilden, Germany) and treated with DNAse I, and cDNA was subsequently synthesized (Transcriptor First Strand cDNA Synthesis kit, Roche, Mannheim, Germany) according to the manufacturers’ instructions. Real-time PCR assays were performed in a final volume of 20 µL, which contained 300 nM of each primer, 2 µL of diluted cDNA sample and 10 µL of Fastart SYBR Green Master Mix (Roche, Mannheim, Germany) using a Mastercycler® ep realplex (Eppendorf, Le Pecq, France). To determine the primer pair efficiency of each gene of interest, a pool of *Quercus petraea* and *Quercus robur* cDNA was used to generate standard curves. For AQP genes, primer sets were carefully designed in the 3′ untranslated region to ensure the amplification of the target gene was specific (for primer sequences, see Table S2). PCR assays were performed in triplicate from a five-fold dilution series. To check for primer dimers, control reactions without cDNA were performed. For each pair of primers, the annealing temperature (*Ta*) and MgCl_2_ concentration were optimized. All PCR reactions were conducted in a 96-well reaction plate using the following parameters: 95°C for 10 min, 40 cycles of 95°C for 15 sec and *Ta* for 30 sec, and 72°C for 15 sec, according to the manufacturer’s instructions. To verify the absence of non-specific products, a melting curve program was applied following PCR amplification. The dissociation program consisted of 95°C for 15 sec, 60°C for 15 sec and a gradual incubation to 95°C over the course of 20 min. The stability of housekeeping genes was determined using Best-Keeper® software (http://www.gene-quantification.de/bestkeeper.html), and elongation factor 1 alpha, cyclophylin and polyubiquitin were used as internal controls [31]. The relative expression of all genes was calculated using the delta-delta-Ct method [32]. The statistical evaluation of data was performed according to the efficiency calibrated model using the REST® program (http://www.gene-quantification.de/rest-2009.html) and assuming that a normal distribution of gene expression was not expected [33].

## Results and Discussion

### Root Hydraulic Conductivity

The whole root system hydraulic conductivity (Lp*r*) was similar for *Quercus petraea* and *Quercus robur* seedlings (Table 1). In this study, Lp*r* values were substantially higher compared to those measured in older oak seedlings [24], which could be due to a less intensive suberization of roots in our growth conditions. The absence of significant difference of Lp*r* is insufficient to conclude to an absence of differences between species for their single root hydraulic architecture. Indeed their different hydraulic behavior in stress conditions were previously reported and suggest that differences could be expected at the root level [23], [34].

**Table 1:** Whole root hydraulic conductivity of oak seedlings.

Treespecie	*N*	A*r* (cm^2^)	P*r* (MPa)	Lp*r*(10^−8^ m^−1^MPa^−1^)
*Quercus* *petraea*	6	105,29±45,24 a	0,63±0,33 a	4,43±1,51 a
*Quercus* *robur*	8	128,85±33,52 a	0,82±0,27 a	4,96±2,42 a

The whole root surface area (A*r*), the steady state root pressures (P*r*), Lp*r* root hydraulic conductivity. Means ± SD. t-tests were used to compare means of independent samples.

### Anatomical Features of the Primary Root of *Quercus Petraea* and *Quercus Robur*

The localization of apoplastic barriers along the root axis is crucial because they influence radial water transport and water uptake [35]. The characterization of these barriers in a precise experimental setup is essential because of the plasticity of root cellular differentiation under various growth conditions [36].

**Figure 1:**
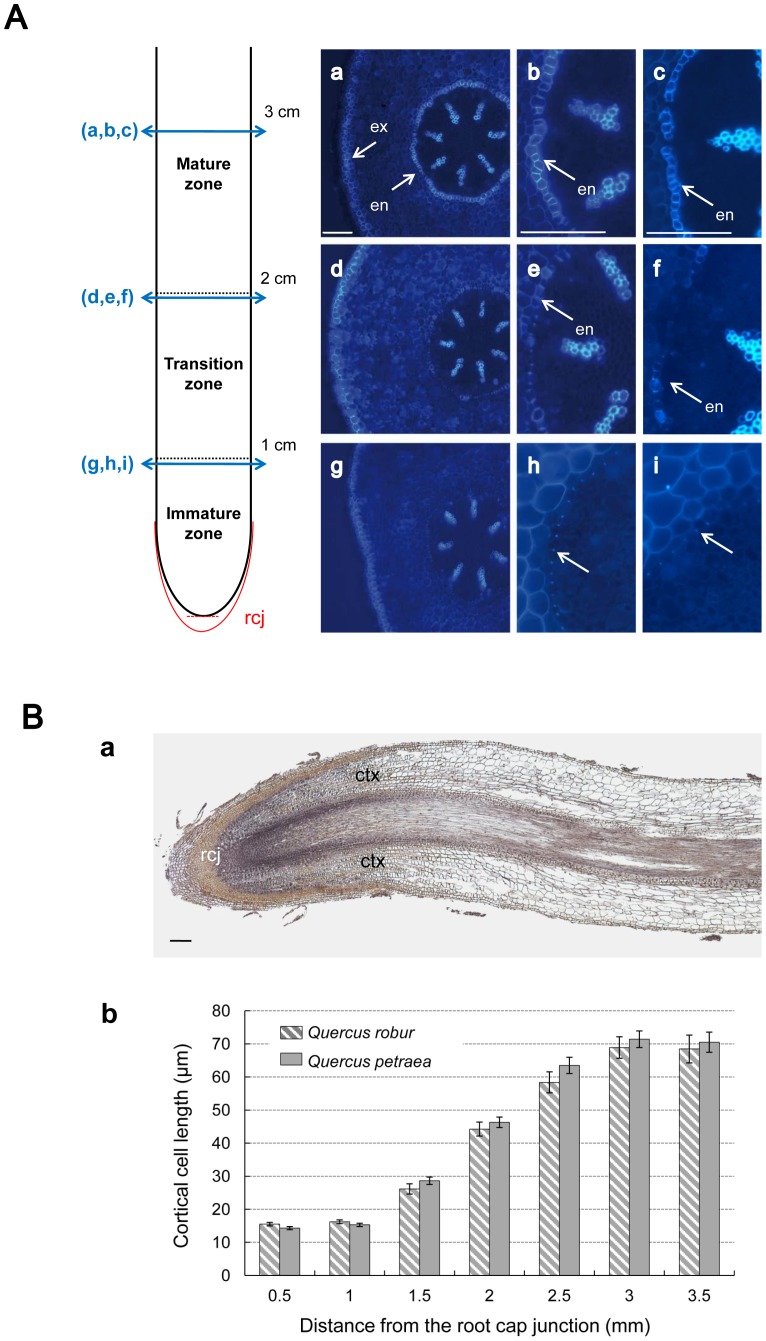
Anatomical features of *Quercus robur* and *Quercus petraea* primary roots. (**A**) Localization of apoplastic barriers along the primary root axis of five-week-old oak seedlings. Vibratome cross-sections through primary roots of *Quercus robur* at 1 cm (g,h), 2 cm (d,e) and 3 cm (a,b) from the root tip stained with berberin hemisulfate. (c,f,i) Detailed view of endodermis differenciation for *Quercus petraea*, at different distance from the root tip. (h,i) Sections through immature zone display discrete dots of fluorescence in the radial walls of endodermal cells (white arrow) which reveals insignificant suberin deposits. (e,f) In the transition zone, the suberization of some endodermal cells (en) was observed. (b,c) Sections cut at 3 cm from the root tip revealed a complete ring of suberized endodermal cells (en). A suberized exodermis (ex) was detected in the three section levels. Scale bars: 100 µm. (**B**) Localization of the zone of cell elongation in oak root tip. (a) A longitudinal root section in immature root zone of *Quercus robur* stained with hematoxylin to measure the length of cortical cells in the root tip. ctx: cortex, rcj: root cap junction. Scale bar: 100 µm. (b) Length of cortical cells as function of distance from the root tip in both oak species. Means ± standard error of the mean (SEM) (n = 4), for each oak species.

Cross sections were cut at a distance of 1, 2, 3 and 4 cm from the root tip for *Quercus robur* and *Quercus petraea* and stained with berberine hemisulfate to visualize suberin and detect the apoplastic barriers [37]. Levels of suberin deposition were very similar between the two species, as presented in Figure 1. At 1 cm from the root tip, small dots of fluorescence (bright blue fluorescent signal) were seen in the innermost layer of the cortex (Figure 1A, h and i). This cell layer corresponds to the future endodermis, an apoplastic barrier crucial for selective water transport from the cortex to the xylem in the root stele. At 2 cm from the root tip, similar small fluorescent dots were observed. However, in this region, the development of Casparian bands was also observed in some endodermal cells for the two oak species (Figure 1A, e and f). We noticed that the deposition of suberin was delayed in the cells facing the xylem poles, which suggests that a lower resistance to radial water flow exists in these root locations. At 3 cm from the root tip, the endodermis was clearly differentiated around the central cylinder, and Casparian bands were visible in most cells (Figure 1A, b and c). Similar results were observed for the cross sections cut at 4 cm from the root tip (data not shown). Strong fluorescence was also observed in the cell walls of the exodermis, which demonstrated that suberin was deposited in all three regions of the root tip. This observation indicates that the apoplastic barrier is consistently present in this tissue along the primary root. The measurement of cortical cell length using longitudinal sections revealed that cell elongation occurs in the 1–3 mm region above the root cap junction (Figure 1B). According to these histological results, three developmental regions can be identified in primary roots. From the root tip, the first 1-cm region corresponds with the immature zone, which includes the regions of mitosis and cell elongation, and it is also characterized by an immature endodermis. The 1-cm region above this first region represents the transition zone and has a partially suberized endodermis. Cross sections of the third and fourth 1-cm regions from the root tip correspond with the mature zone, which has a suberized endodermis and exodermis. Similar levels of endodermal differentiation were observed for the two oak species. However, this result does not exclude differences in the composition or arrangement of suberin polymers, which can determine the permeability efficiency of root apoplastic barriers [38]. Previous studies have found variable levels of suberized endodermis in the primary root tip of *Quercus suber* and *Quercus robur*, depending on the oak species and environmental growth conditions [36], [39].

### Structure and Phylogenetic Analysis of Isolated AQPs

**Figure 2:**
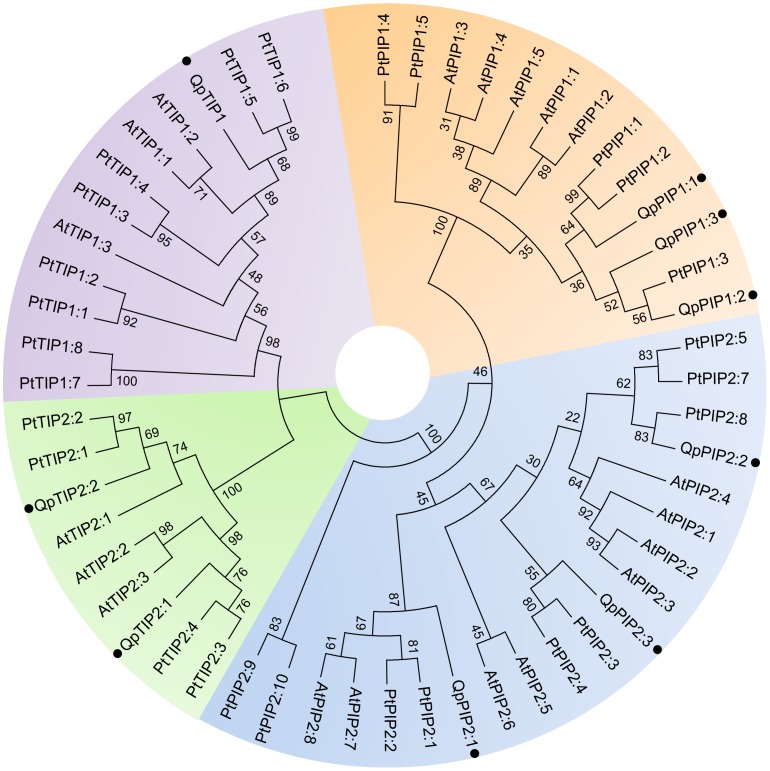
Phylogenetic analysis of oak AQP proteins. Phylogenic tree showing the four clusters PIP1, PIP2, TIP1 and TIP2. The nine *Quercus petraea* AQPs are compared with all the PIPs as well as all the TIP1s and TIP2s from *Arabidopsis thaliana* and *Populus trichocarpa.* Maximum likehood phylogenetic analysis and bootstrap test were performed using MEGA 5. Identified subgroups are indicated by different colors and oak AQP names are marked by full circles. Branch lengths are proportional to evolutionary distance.

In this study, we isolated nine full-length cDNAs encoding putative AQPs in *Quercus petraea* using PCR cloning. The resulting data, including the gene names, accession numbers, length of coding regions and deduced polypeptides, are summarized in Table 2. Using the BLASTP and BLASTX programs, a comparison of isolated oak sequences and those of other plants revealed a high sequence identity at protein level (84–94%) and at nucleotide level (63–84%) (Table S5). The open reading frames of AQP cDNA clones were predicted to encode polypeptides that were 248–289 amino acids in length (Table 2), with sequence identities of 25–91% (Table S6). Six of the identified sequences were highly similar to PIP sequences, whereas the remaining three sequences showed homology to TIP sequences. Based on the predicted amino acid sequences, a phylogenetic analysis confirmed that the oak AQPs could be classified into two orthologous groups of PIPs and TIPs (Figure 2 and Figure S2). The TIP subfamily exhibits two distinct clusters corresponding to the TIP1 and TIP2 subgroups known to have different cellular localizations and functions [40]. The PIP subfamily is divided into the PIP1 and PIP2 subgroups but has a lower pairwise-sequence divergence compared with the TIP subfamily. This observation might suggest a slower rate of evolution of PIPs compared with TIPs [41]. Because of the high amino-acid sequence homology and functional diversity of MIPs in plants, a thorough analysis of signature sequences and residues in discriminant positions is required to precisely identify and classify newly isolated AQP genes.

**Table 2:** Summary of oak *AQP* characteristics.

cDNA name	GenBank accession No.	ORF (bp)	Protein length (aa)	Highest similarity in other plants (%)
PIP2;1	JQ846268	834	278	*Vitis vinifera*, ABH09327 (94)
PIP2;2	JQ846269	855	285	*Populus trichocarpa*, ABK94847 (90)
PIP2;3	JQ846270	858	286	*Pyrus communis*, BAB40143 (90)
PIP1;1	JQ846271	867	289	*Juglans regia*, ACR56611 (92)
PIP1;2	JQ846272	858	286	*Gossypium hirsutum*, ABD63904 (92)
PIP1;3	JQ846273	858	286	*Gossypium hirsutum*, ABD63904 (92)
TIP1	JQ846274	750	250	*Vitis vinifera*, ABH09330 (93)
TIP2;1	JQ846275	744	248	*Solanum tuberosum*, AAB67881 (84)
TIP2;2	JQ846276	753	251	*Malus prunifolia*, AEQ29858 (90)

The open reading frame and protein length are detailed for all genes identified in this study. The highest sequence identity between the predicted amino acid sequences of oak AQPs and those of other plants was determined using BLASTP (http://blast.ncbi.nlm.nih.gov/Blast.cgi). The parentheses indicate the percentage of sequence identity at the amino acid level.

**Figure 3:**
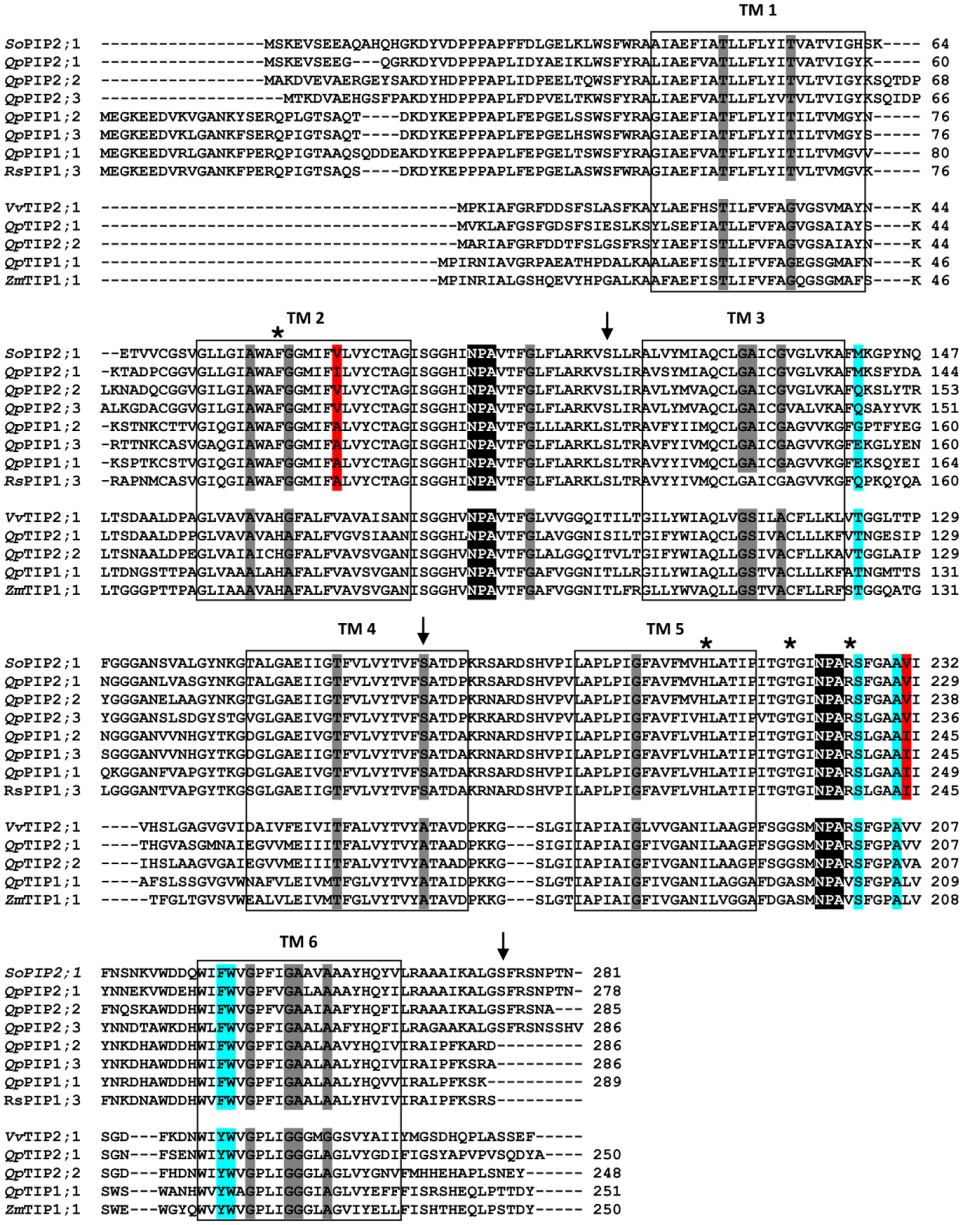
Comparative alignment of predicted amino acid sequences isolated from *Quercus petraea* and representative AQPs in plants. The four AQPs *So*PIP2;1 (AAA99274.2), *Rs*PIP1;3 (BAA92259.1), *Vv*TnTIP2;1 [46] and *Zm*TIP1;1 (NP_001104896.1) have been functionally characterized as water channels. Black boxes represent predicted transmembrane helices and the AQP NPA sequence signature is underlined in black. The conserved amino acid typically found in the constriction region of the pore (Ar/R filter) are indicated by stars, and conserved residues located at Froger’s positions are shaded in blue. Amino acids identified for being involved in specificity of water transport are shaded in red. Serine residues, appointed by arrows, are a component of plant AQP gating and residues occurring in the helix-helix interfaces are underlined in dark grey.

In our study, both the TMpred and OCTOPUS analyses predicted a topological model for the deduced protein sequences that consisted of six alpha-helical transmembrane helices (TM1 to TM6, Figure 3) connected to five loops with the N and C terminal ends located on the cytoplasmic side of the membrane (Figure S3). These results agree with the reported topology of AQPs [42]. In addition, the neural network method of OCTOPUS detected two small peaks between TM2 and TM3 and between TM5 and TM6, which revealed the presence of two additional minor helices. These small helical regions are of particular importance for AQP structure because they contain the conserved NPA (asparagine-proline-alanine) motifs that are functionally important for the major constriction of water channels. Figure 3 shows the alignment of deduced amino acid sequences of oak and some well-described AQPs in plants [43], [44], [45], [46]. Small and weakly polar residues are found in the helix-helix interfaces and conserved among the nine AQPs (Figure 3), as previously observed in other plants [8], [9], [47]. The aromatic/Arginine (ar/R) selectivity filter is particularly important for AQP function because it limits solute permeability. Some residues of the oak amino acid sequences (Table 3) were similar to those found in *Populus trichocarpa* and other characterized AQPs of herbaceous plant models [8], [9], [47]. The Ar/R filters harbored identical residues for all isolated PIPs, including a Phe (H2 position), His (H5 position) and Arg (LE2 position) (Table 3), which are typical of water-specific AQP structures [47]. The His and Arg residues might provide donor hydrogen bonds for water molecules. A conserved Ala/Ile (Val) residue differed between the PIP2s and PIP1s (Table 3). PIP1s had an Ala residue, whereas PIP2s had a Val or Ile. In *Oryza sativa*, this residue was found to be involved in the osmotic water permeability of AQPs [48]. The presence of a Val or Ile residue in helix 2 conferred a high permeability to water of PIP2 members, compared to PIP1 members exhibiting a Ala residue at this location. Site-directed mutation of Ile^244^ with Val increased the water permeability of PIP1;3 in radish [44]. Functional studies of AQPs revealed that PIP2s exhibit a high osmotic water permeability in contrast with PIP1 members that show lower or no water permeability when expressed in *Xenopus* oocytes in maize [49], [50], poplar [51], gravepine [52] and wheat [53], or when expressed in lily pollen protoplasts in *Arabidopsis thaliana* [54].

**Table 3:** Conserved amino acid residues of isolated oak AQPs.

	Ar/R selectivity filters				Froger’s positions					Ala/Ile/Val residues	
Aquaporin gene	H2	H5	LE1	LE2	P1	P2	P3	P4	P5	TM2	Loop E
PIP2;1	F	H	T	R	M	S	A	F	W	I	V
PIP2;2	F	H	T	R	Q	S	A	F	W	V	V
PIP2;3	F	H	T	R	Q	S	A	F	W	V	V
PIP1;1	F	H	T	R	E	S	A	F	W	A	I
PIP1;2	F	H	T	R	G	S	A	F	W	A	I
PIP1;3	F	H	T	R	E	S	A	F	W	A	I
TIP1	H	I	A	V	T	S	A	Y	W	–	–
TIP2;1	H	I	G	R	T	S	A	Y	W	–	–
TIP2;2	H	I	G	R	T	S	A	Y	W	–	–

For this subfamily, the Ar/R selectivity filters (H2, H5, LE1 and LE2) and Froger’s positions (P1–P5) are given for all AQPs, and the variable Ala/Ile/Val residues identified in the PIPs as involved in water permeability is indicated.

Isolated TIPs showed greater diversity within the putative pore regions because two different ar/R subgroups had different residues in the LE1 and LE2 positions (Table 3). From this observation, we identified one TIP1 and two TIP2s in *Quercus petraea*. Five conserved residues (P1–P5 positions) were previously identified by Froger et al. (1998) [55] and provide functional specificities that differ between orthodox AQPs and aquaglyceroporins. The oak sequences harbored similar residues in the P2 to P5 positions, with a S (Ser)- A (Ala) pair at the P2 and P3 positions and F (Phe)- W (Trp) pair of aromatic residues at the P4 and P5 positions, which is generally observed in orthodox AQPs. The importance of these residues for water permeability has been functionally demonstrated [56] and suggests that the isolated, putative TIPs are expected to exhibit similar functional properties when compared to the putative PIPs.

Based on phylogenetic analysis and sequence homology, the identified oak AQP genes were named according to the standard nomenclature for MIPs. The isolated genes were classified as 3 PIP2s (*PIP2;1*, *PIP2;2* and *PIP2;3*), 3 PIP1s (*PIP1;1*, *PIP1;2* and *PIP1;3*), 1 TIP1 and 2 TIP2s (*TIP2;1* and *TIP2;2*). The presence of residues at amino acids involved in the specificity of water transport, including the P4–P5 residues at Froger’s positions, ar/R filter residues and conserved NPA domains, suggests that the identified, putative oak AQPs exhibit water channel activity.

### Differential Transcript Abundance Among *Quercus petraea* and *Quercus robur* AQPs in the Primary Root

**Figure 4:**
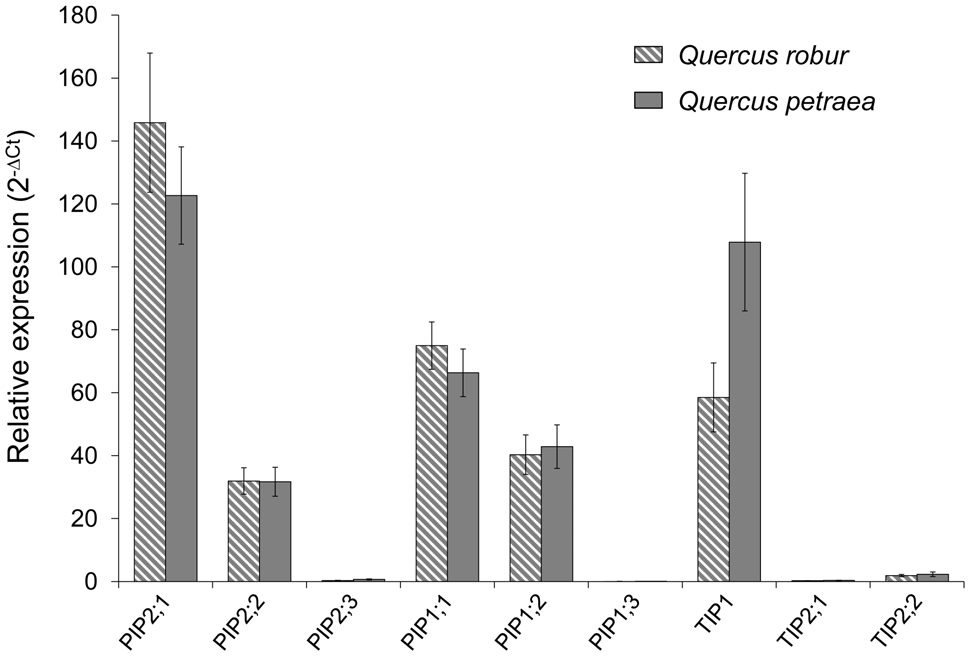
AQP transcript abundance in primary roots of *Quercus robur* and *Quercus petraea*. Expression analysis was determined from 21 independent RNA preparations. Means ± standard error of the mean (SEM) (n = 21).

The gene expression levels of the AQPs were measured using real-time PCR. In both species, *PIP2;1*, *PIP1;1*, *PIP1;2*, *PIP2;2* and *TIP1* were the highly expressed AQPs, and *PIP2;1* was the most abundant gene in *Quercus robur* (Figure 4).

**Figure 5:**
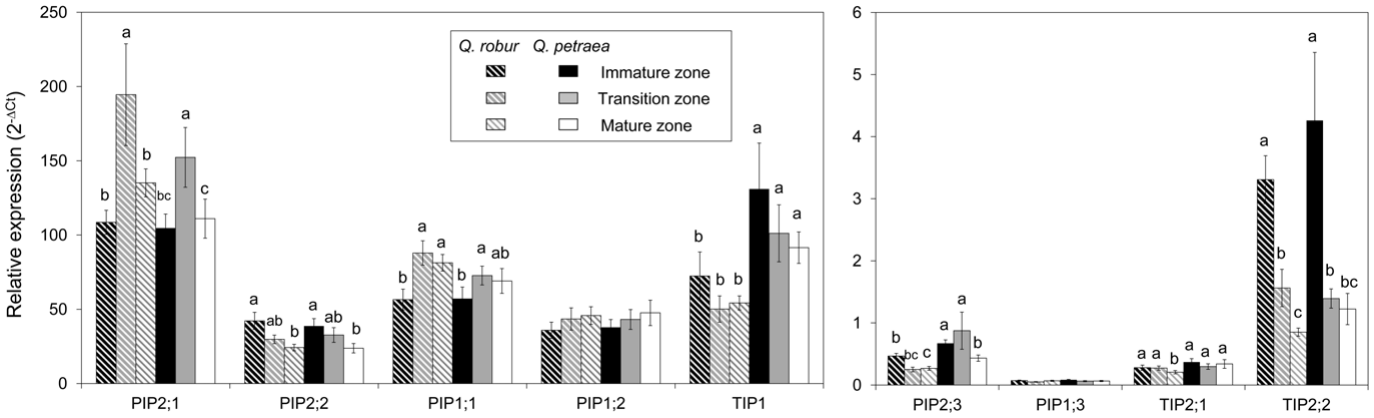
AQP transcript abundance in the developmental primary root zones of *Quercus robur* and *Quercus petraea*. The relative expression of AQPs was measured in different root segments from the root tip of the immature, transition, and mature zones. For each root zone, expression analysis was performed from 21 RNA extractions, which corresponded with 21 batches of three oak seedlings. Means ± SEM (n = 21). Significant differences are indicated by the letters a, b and c.

Of the nine AQP genes tested, *PIP2;1*, *PIP2;3*, *TIP1*, and *TIP2;1* exhibited differential gene expression between the two oak species in the three root zones (Figure 5). The transcript abundance of *PIP2;1* and *TIP2;1* was significantly different between the two species in the mature zone (P = 0.044 and P = 0.015, respectively). However, the relative expression of these genes was higher but not significant in both the immature and transition zones. *Quercus petraea* displayed a higher transcript abundance of *PIP2;3* and *TIP1* compared with *Quercus robur* in the three developmental root zones (P = 0.007 and P<0.001, respectively). *TIP1* is abundant in oaks, as previously reported for some *TIP1* genes in *Zea mays* and *Hordeum vulgare* roots [57], [58]. Several studies evidenced that TIPs can regulate water transport at cellular level. In particular, certain TIP isoforms are involved in osmotic regulation between the vacuole and cytoplasm and exhibit water channeling activity [59]. However, the insertional inactivation of *AtTIP1;1* did not demonstrate a crucial role for this gene in water flow at the whole plant level [20].

Of the nine oak AQPs tested, six AQPs exhibited variable expression along the primary root axis (Figure 5). The relative abundance of *PIP2;2*, *PIP2;3* and *TIP2;2* mRNAs decreased in the mature zone with respect to the immature zone, which suggests that these genes are important for water transport in the immature zone. *TIP* expression was previously reported to be developmentally regulated in *Arabidopsis thaliana* roots and generate fusions of all TIP complete sequences [60]. In particular, *AtTIP4;1*, which is root-specific, has been reported to be developmentally regulated because this gene exhibited a high expression level in the differentiation and elongation zones and lower expression levels as the root matured. Another study in this plant model reported that none of the TIP isoforms tested were expressed in the meristematic region of the root [61]. These observations suggest that a subset of TIPs is specifically involved in root cell elongation. Some TIP members are highly selective AQPs, and confer a higher water permeability of the tonoplast compared to the plasma membrane [62]. It is hypothesized that these AQPs may allow a rapid osmotic equilibration between the cytoplasmic and vacuolar compartments during the cell elongation process [63].

In *Quercus petraea*, *PIP2;1* and *PIP1;1* display a higher expression level in the transition zone compared with the immature zone (Figure 5, P = 0.039 and P = 0.018, respectively). Similar patterns were found in *Quercus robur* for *PIP2;1* and *PIP1;1* (P = 0.019 and P = 0.005, respectively). These results are in agreement with cell-specific expression of mRNAs in different root tissues, including the cortex, endodermis and epidermal atrichoblasts, evidenced at three developmental stages in the *Arabidopsis thaliana* primary root [64]. The four most abundant PIPs tested, i.e., *AtPIP2;1*, *AtPIP2;3*, *AtPIP1;1* and *AtPIP1;2*, exhibited a higher expression level in the differentiation zone compared with the immature zone. In *Zea mays*, a previous study highlighted two predominant isoforms named *Zm*PIP1;5 and *Zm*PIP2;5 in the differentiated primary root zone [65]. A decrease in symplastic continuity between cells resulted in a general increase in *ZmPIP* transcripts along the elongation and mature zones to maintain water transport through the plasma membrane. In our study, *PIP2;1* and *PIP1;1* were abundant genes in both oak species and exhibited a higher expression level in the mature zone compared with the immature zone in *Quercus robur* (Figure 5, P = 0.039 and P = 0.005, respectively). Variations in the expression of these genes along the root axis differ between oaks, and reveal differences in transcriptional control for *Quercus robur* and *Quercus petraea* according to tissue differentiation. This observation opens an interesting perspective in understanding processes involved in radial water conductance in both species.

In this paper, we report the first characterization of the expression of nine AQPs in the primary root axis of *Quercus petraea* and *Quercus robur*. Four AQP genes, *PIP2;1*, *PIP2;3*, *TIP2;1* and *TIP1*, were highlighted because of their significantly different relative expression between the two oak species in the different developmental root zones. In particular, *PIP2;1* is an abundant gene, and exhibit a differential expression between the two oaks and variable expression along the root axis. Further elucidation of the role of individual AQP genes in root water transport will facilitate the determination of how specific AQP members contribute to the contrasting tolerance of *Quercus petraea* and *Quercus robur* to stress conditions and natural distribution of these species.

## Supporting Information

Figure S1 **Multiple sequence alignment of the predicted amino acid sequences of** ***Quercus petraea*** **and** ***Quercus robur*** **.** The sequences were identified from SSH libraries with a representative AQP sequence of *Olea europea* from the PIP2 (a), PIP1 (b) and TIP (c) subfamilies. The GenBank accession numbers of the protein sequences are as follows: *Oe*PIP2;1: DQ202709, *Oe*PIP1;1: DQ202708 and *Oe*TIP1;1: DQ202710. Canonical NPA-NPA motifs are underlined in black. Sequence homology between the oak sequences is indicated by green boxes, and amino acids shown in red represent residues that varied between several predicted amino acid sequences.
(DOC)

Figure S2 **Molecular phylogeny of the oak AQPs.** Deduced amino acid sequences from *Quercus petraea* and (a) sequences of *Arabidopsis thaliana* or (b) sequences of *Populus trichocarpa* were used to construct the tree. Maximum likehood phylogenetic analysis and bootstrap test were performed using MEGA 5. Vertical black bars indicate identified subgroups and oak AQP names are showed in color. Branch lengths are proportional to evolutionary distance.
(DOC)

Figure S3 **Oak AQP results from the TMpred (1) and OCTOPUS (2) servers.** PIP2;1 (a), PIP2;2 (b), PIP2;3 (c), PIP1;1 (d), PIP1;2 (e), PIP1;3 (f), TIP2;1 (g), TIP2;2 (h) and TIP1 (i). Helical, membrane-spanning present peaks corresponding with the six major transmembrane domains (indicated by red lines) were predicted using TMpred. Details regarding the length and position of the transmembrane regions of the amino acid sequence are provided in the accompanying table. The presence of six transmembrane helices, marked in red, was confirmed from topology predicted using OCTOPUS and is shown in the upper schematic of the AQP topology. The green and brown loops are predicted to be located in the cytosolic and extracellular parts, respectively. The two additional red peaks in the middle graph correspond with minor helices, which were found for all oak AQPs (denoted by black arrows).
(DOC)

Table S1 **The list of mRNA sequences identified from SSH libraries (a) and details of their use in RACE experiments (b).**
(DOC)

Table S2 **Details of the real-time PCR procedure, including a list of the primers (a) and PCR conditions (b) used in the experiments.**
(DOC)

Table S3 **List of sequences used for phylogenetic analysis.**
(DOC)

Table S4 **Details of the six housekeeping genes tested in primary root zones.** The sequences were aligned using ClustalW (http://www.ebi.ac.uk/Tools/msa/clustalw2/), and a consensus nucleotide sequence was deduced. The reconstituted sequences account for nucleotide variation, which is likely due to nucleotide variation between *Quercus petraea* and *Quercus robur* or sequencing errors. Sequences are reported in the table. Coding regions are indicated in blue, and nucleotide variation is indicated in red. The parentheses denote predictions for specific nucleotides.
(XLS)

Table S5 **BLASTX results for oak AQPs.** cDNAs were compared to cDNAs of other plants. Total score, query coverage and e values are reported.
(XLS)

Table S6 **Protein sequence homology between oak AQPs based on isolated cDNAs.** Sequence homology was determined using ClustalW (http://www.ebi.ac.uk/Tools/msa/clustalw2/).
(DOC)
