# Supplementary material for: Identification and Expression of Nine Oak Aquaporin Genes in the Primary Root Axis of Two Oak Species, Quercus petraea and Quercus robur
Source: PLoS One. 2012 Dec 17;7(12):e51838. doi: 10.1371/journal.pone.0051838 (PMC3524086; doi:10.1371/journal.pone.0051838)
Supplement: Figure S1 — Multiple sequence alignment of the predicted amino acid sequences of Quercus petraea and Quercus robur . The sequences were identified from SSH libraries with a representative AQP sequence of Olea europea from the PIP2 (a), PIP1 (b) and TIP (c) subfamilies. The GenBank accession numbers of the protein sequences are as follows: OePIP2;1: DQ202709, OePIP1;1: DQ202708 and OeTIP1;1: DQ202710. Canonical NPA-NPA motifs are underlined in black. Sequence homology between the oak sequences is indicated by green boxes, and amino acids shown in red represent residues that varied between several predicted amino acid sequences. (DOC) [file pone.0051838.s001.doc]

**Figure S1**

a)

**CU639847 --------VAEHGSFPAKDYHDPPPAPLFDPVELTKWSFYRALIAEFVATLLFLYVTVLTVIGYKSQIDPALKGDACG 43**

**CU656772 ------------------------------------------------------------------------------**

**CU657344 ------------------------------------------------------------------------------**

**PIP2_Olea MTKDVESHVEPAEEYSAKDYHDPPPAPLIDFDELSSWSFYRALIAEFVATLLFLYVTVLTVIGYKSQIDPAHGGDDCG 50**

**CU656754 ------------------------------------------------------------------------------**

**CU656755 ------------------------------------------------------------------------------**

**CU639847 GVGILGIAWAFGGMIFVLVYCTAGISGGHINPAVTFGLFFLARGCVGG------------------------------ 76**

**CU656772 -------------MIFVLVYCTAGISGGHINPAVTFGLFLARK----------------------------------- 20**

**CU657344 --------WAFGGMIFILVYRTAGISGGHINPAVTFGLFLARKVSLIRAVSYMIAQCLGAICGVGLVKAFMKSFYDAN 49**

**PIP2_Olea GVGILGIAWAFGGMIFILVYCTAGISGGHINPAVTLGLFLGRKVSLIRAVLYMVAQCLGAICGVGLVKAFQKSYYHKY 100**

**CU656754 ------------------------------------------------------------------------------**

**CU656755 ------------------------------------------------------------------------------**

**CU639847 ------------------------------------------------------------------------------ 76**

**CU656772 ------------------------------------------------------------------------------ 20**

**CU657344 GGGANLVASGYNKGTALGAEIIGTF----------------------------------------------------- 68**

**PIP2_Olea GGGANELAPGYNKGVGLGAEIIGTFVLVYTVFSATDPKRNARDSHVPVLAPLPIGFAVFMVHLATIPITGTGINPARS 150**

**CU656754 -----------------------------------------------------------------------GINPARS 7**

**CU656755 ---------------------------------------------------------------------GTGINPARS 9**

**CU639847 ----------------------------------------------------- 76**

**CU656772 ----------------------------------------------------- 20**

**CU657344 ----------------------------------------------------- 68**

**PIP2_Olea FGAAVIYNEDKPWDDHWIFWVGPFVGALIAAIYHQYILRAGAIKALGSFRSNA 186**

**CU656754 FGAAVIFNQSKAWDDHWIFWVGPFVGAAIAAFYHQFILRAAAIKALGSFRSNA 43**

**CU656755 FGAAVIFNQSKAWDDHWIFWVGPFVGAAIAAFYHQFILRAAAIKALGSFRSNA 45**

**b)**

**CU657358 ----------KFPESKPIGTAAQSQDDEAKDYKEPPPAPLFEPGALTSWSFYRAGTAEFVATFLFLYITILTVMGVVK 40**

**CU639694 MEGKEEDVKLGANKFSERQPIGTSAQ??DKDYKEPPPAPLFEPGELHSWSFWRAGIAEFIATFLFLYITILTVMGYSR 41**

**CU639735 ------------------------------------------------------------------------------**

**CU656596 ------------------------------------------------------------------------------**

**CU656421 ------------------------------------------------------------------------------ CU656415 ------------------------------------------------------------------------------**

**CU656915 ------------------------------------------------------------------------------**

**PIP1_Olea MESKEEDVRVGANKFAERQPIGTAAQSQDKDYKEPPPAPLFEPGELSSWSFYRAGIAEFIATFLFLYITVLTVMGVSK 45 CU656237 ------------------------------------------------------------------------------**

**CU657358 SPTKCSTVGIQGIAWAFGGMIFALVYC--------------------------------------------------- 59 CU639694 TTNKCASVGAQGIAWAFGGMIFALVYCTAGISGGHINPAVTFGLFLARKLSLTRAVFYIVMQCLGAICGAGVVKG-EK 87**

**CU639735 --------------------------------GGHINPAVTFGLLLARKLSLTRAVFYMIMQCLGAICGAAVVKGFQK 31**

**CU656596 ------------------------------------------------------------------------------ CU656421 ------------------------------------------------------------------------------ CU656415 ------------------------------------------------------------------------------**

**CU656915 ------------------------------------------------------------------------------ PIP1_Olea SDSKCKTVGIQGIAWAFGGMIFALVYCTAGISGGHINPAVTFGLFLGRKLSLTRAVFYMVMQCLGAICGAGVVKGFGK 68 CU656237 -------------------MIFALVYCTAGISGGHINPAVTFGLLLARKLSLTRAVFYIIMQCLGAICGAGVVKGFGP 39**

**CU657358 ------------------------------------------------------------------------------ 59 CU639694 ------------------------------------------------------------------------------ 87 CU639735 NQYINILWHHIIRCYSGLQKNG-------------------------------------------------------- 45**

**CU656596 ------------------------------FVLVYTVFSATDAKRSARDSHVPILAPLPIGFAVFLVHLATIPITGTG 31**

**CU656421 ------------------------------FVLVYTVFSATDAKRSARDSHVPILAPLPIGFAVFLVHLATIPITGTG 31**

**CU656415 ------------------------AARAGTFVLVYTVFSATDAKRSARDSHVPILAPLPIGFAVFLVHLATIPITGTG 37**

**CU656915 --------------------------RAGTFVLVYTVFSATDAKRSARDSHVPILAPLPIGFAVFLVHLATIPITGTG 35**

**PIP1_Olea TLYMTKGGGANVVAHGYTKGDGLGAEIIGTFVLVYTVFSATDAKRSARDSHVPILAPLPIGFAVFLVHLATIPITGTG 111**

**CU656237 TFYEG------------------------------------------------------------------------- 44**

**CU657358 ---------------------------------------------------- 59 CU639694 ---------------------------------------------------- 87 CU639735 ---------------------------------------------------- 45**

**CU656596 INPARSLGAAIIYNRDHAWDDQWIFWVGPFIGAALAALYHQVVIRALPFKSK 65**

**CU656421 INPARSLGAAIIYNRDHAWDDQWIFWVGPFIGAALAALYHQVVIRALPFKSK 65**

**CU656415 INPARSLGAAIIYNRDHAWDDQWIFWVGPFIGAALAALYHQVVIRALPFKSK 70**

**CU656915 INPARSLGAAIIYNRDHAWDDQWIFWVGPFIGAALAALYHQVVI-------- 64**

**PIP1_Olea INPARSLGAAIIYNKDKAWDDHWIFWVGPFIGAALAALYHVVVIRAIPFKK- 146**

**CU656237 ---------------------------------------------------- 44**

c)

**CU656921 MPISQIAIGSPAEFGQADSLKAALAEFISVLIFVFAGEGSGMAFNKLTDDGSTTPAGLVAAALAHAFALFVAVAIGAN**

**CU656541 MPISQIAIGSPAEFGQADSLKAALAEFISVLIFVFAGEGSGMAFNKLTDDGSTTPAGLVAAALAHAFALFVAVAIGAN**

**CU640246 --MARIAFGRFDDTFSLGSFRSYIAEFISTLLFVFAGVGSAIAYNKLTSNAALDPEGLVAIAICHGFALFVAVSVGAN**

**CU656364 --MARIAFGRFDDSFSLGSFKSYIAEFISTLLFVFAGVGSAIAYNKLTSNAALDPEGLVAIAICHGFALFVAVSVGAN**

**CU656728 ------------------------------------------------------------------------------**

**TIP_Olea MPISRVAIGSPAEFSQLDAIKAAVAEFISMLIFVFAGQGSGMAFAKLTDNESTTPAGLIAASIAHAFALFVAVSVGAN**

**CU640266 ------------------------------------------------------------------------------**

**CU640067 ------------------------------------------------------------------------------**

**CU656131 ------------------------------------------------------------------------------**

**CU656921 ISGGHVNPAVTFGAFLGGHISLIRGILYLPG-----------------------------------------------**

**CU656541 ISGGHVNPAVTFGAFLGGHISLI-------------------------------------------------------**

**CU640246 ISGGHVNPAVTFGLALGGQITVLTGIFYWIAQLLGSIVACFLLKAVTGGLAIPIHSLAAGVGAIEGVVMEIIITFALV**

**CU656364 ISGGHVNPAVTFGLALGGQITV--------------------------------------------------------**

**CU656728 ------------------------------------------------------------------------------**

**TIP_Olea ISGGHVNPAVTLGAFVGGHITLFRSIMYWIAQLLGSVIACLLLKFATGGLETSAFALSSGVTVWNAVIFEIVMTFGLV**

**CU640266 ------------------------------------------------------------------VVMEIIITFALV**

**CU640067 ------------------------------------------------------------------VVMEIIITFALV**

**CU656131 ----------------------------------------------------------AWSRPRYAGVMEIIITFALV**

**CU656921 ------------------------------------------------------------------------------**

**CU656541 ------------------------------------------------------------------------------**

**CU640246 YTVYAT------------------------------------------------------------------------**

**CU656364 ------------------------------------------------------------------------------**

**CU656728 ----ATAIDPKKGSLGTIAPIAIGFIVGANILAGGAFDGASMNPAVSFGPALVSWSWANHWVYWAGPLIGGGIAVLWV**

**TIP_Olea YTVYATAVDPKKGNLGIIAPIAIGFIVGANILAGGAFDGASMNPAVSFGPAVVSWTWDSHWVYWLGPFVGAGIAALVY**

**CU640266 YTVYATAADPKKGSIGIIAPIAIGFIVGANILAAGPFSGGSMNPARPFGPAVVSGNFSENWIYWVGPLIGGGLAGLV-**

**CU640067 YTVYATAADPKKGSIGIIAPIAIGFIVGANILAAGPFSGGSMNPARSFGPAVVSGNFSENWIYWVGPLIGGGLAGLVY**

**CU656131 YTVYATAADPKKGSIGIIAPIAIGFIVGANILAAGPFSGGSMNPARSFGPAVVSGNFSENWIYWVGPLIGGGLAGLVY**

**CU656921 ------------------**

**CU656541 ------------------**

**CU640246 ------------------**

**CU656364 ------------------**

**CU656728 ------------------**

**TIP_Olea EILFINQSHQQLPTSAEY**

**CU640266 ------------------**

**CU640067 GDIFIGSYAPVPVSQDYA**

**CU656131 GDIFIGSYAPVPVSQDYA**
