# Supplementary figures and images for: Identification and Expression of Nine Oak Aquaporin Genes in the Primary Root Axis of Two Oak Species, Quercus petraea and Quercus robur
Source: PLoS One. 2012 Dec 17;7(12):e51838. doi: 10.1371/journal.pone.0051838 (PMC3524086; doi:10.1371/journal.pone.0051838)

**Figure S2**

**a)**

**
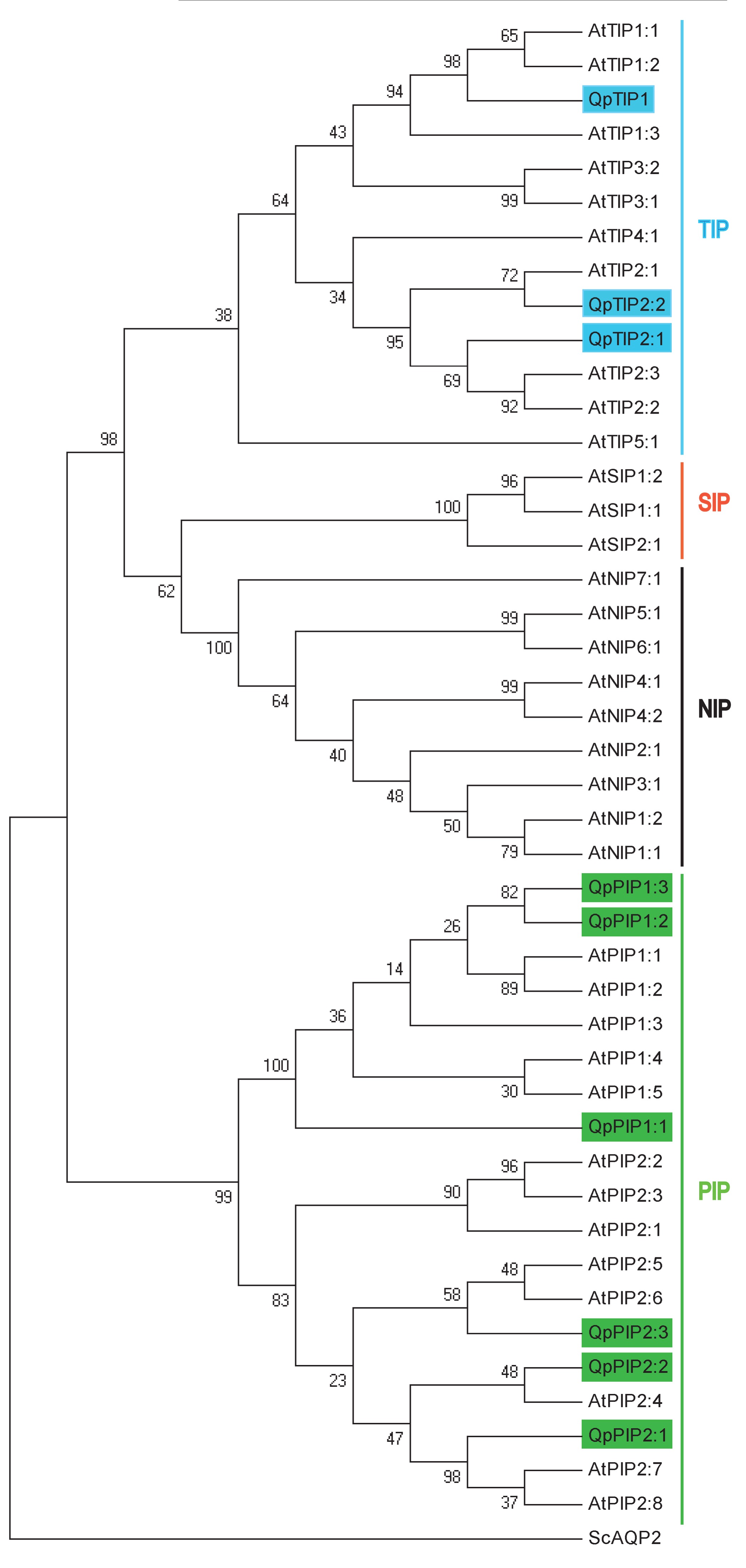
**

**b)**

**
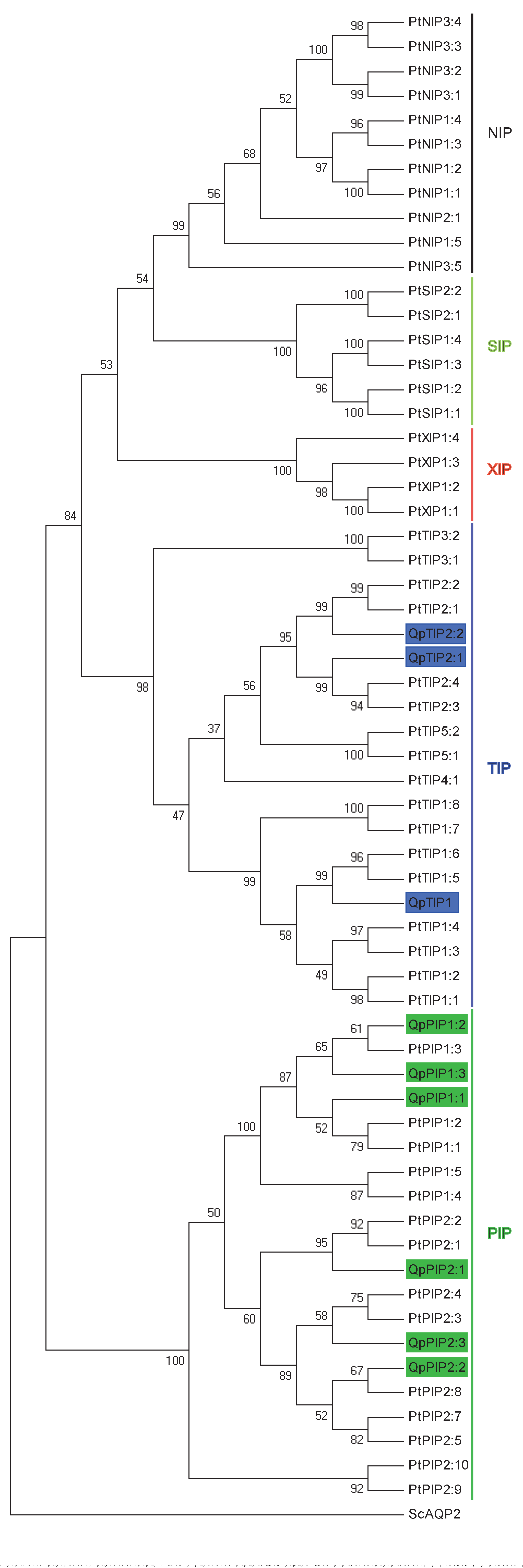
**

Supplement: Figure S2 — Molecular phylogeny of the oak AQPs. Deduced amino acid sequences from Quercus petraea and (a) sequences of Arabidopsis thaliana or (b) sequences of Populus trichocarpa were used to construct the tree. Maximum likehood phylogenetic analysis and bootstrap test were performed using MEGA 5. Vertical black bars indicate identified subgroups and oak AQP names are showed in color. Branch lengths are proportional to evolutionary distance. (DOC) [file pone.0051838.s002.doc]
