# Supplementary material for: Identification and Expression of Nine Oak Aquaporin Genes in the Primary Root Axis of Two Oak Species, Quercus petraea and Quercus robur
Source: PLoS One. 2012 Dec 17;7(12):e51838. doi: 10.1371/journal.pone.0051838 (PMC3524086; doi:10.1371/journal.pone.0051838)

(1)

(a)


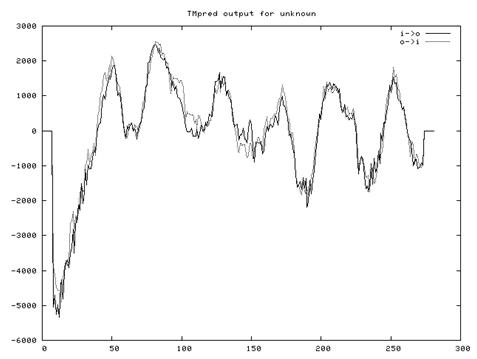


| **Transmembrane helice** | **from** | **to** | **length** | **score** |
| --- | --- | --- | --- | --- |
| **1** | 41 | 63 | 23 | 1885 |
| **2** | 74 | 90 | 17 | 2553 |
| **3** | 120 | 138 | 19 | 1660 |
| **4** | 165 | 181 | 17 | 1332 |
| **5** | 197 | 219 | 23 | 1391 |
| **6** | 244 | 261 | 18 | 1815 |

(b)


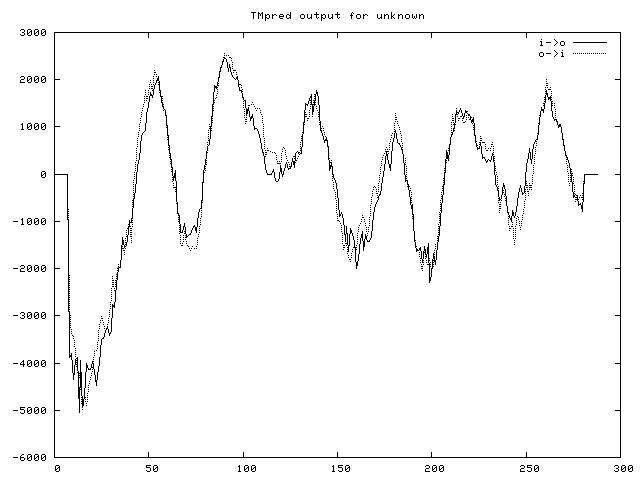


| **Transmembrane helice** | **from** | **to** | **length** | **score** |
| --- | --- | --- | --- | --- |
| **1** | 44 | 66 | 23 | 2059 |
| **2** | 81 | 99 | 19 | 2555 |
| **3** | 129 | 150 | 22 | 1767 |
| **4** | 174 | 190 | 17 | 1292 |
| **5** | 206 | 228 | 23 | 1391 |
| **6** | 253 | 270 | 18 | 2008 |

(c)


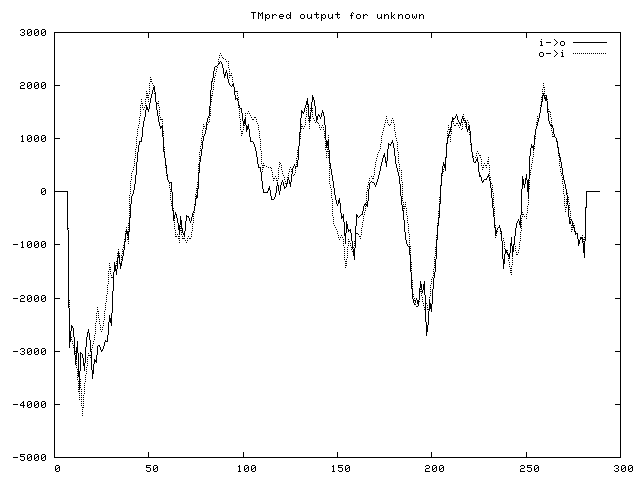


| **Transmembrane helice** | **from** | **to** | **length** | **score** |
| --- | --- | --- | --- | --- |
| **1** | 42 | 64 | 23 | 1988 |
| **2** | 81 | 97 | 19 | 2621 |
| **3** | 127 | 148 | 22 | 1816 |
| **4** | 169 | 185 | 17 | 1418 |
| **5** | 204 | 226 | 23 | 1448 |
| **6** | 251 | 268 | 18 | 2044 |

(d)


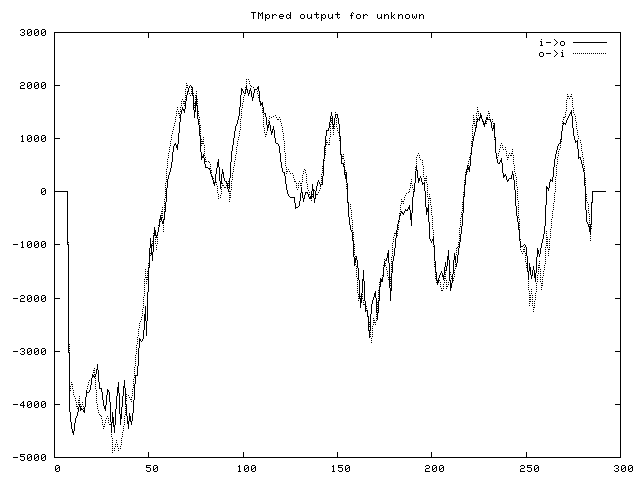


| **Transmembrane helice** | **from** | **to** | **length** | **score** |
| --- | --- | --- | --- | --- |
| **1** | 68 | 84 | 22 | 2002 |
| **2** | 95 | 112 | 18 | 2123 |
| **3** | 140 | 158 | 19 | 1498 |
| **4** | 185 | 206 | 22 | 725 |
| **5** | 217 | 239 | 23 | 1451 |
| **6** | 264 | 283 | 20 | 1854 |

(e)


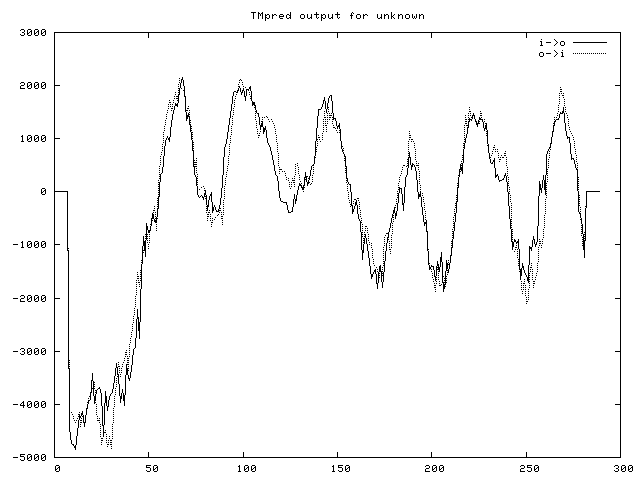


| **Transmembrane helice** | **from** | **to** | **length** | **score** |
| --- | --- | --- | --- | --- |
| **1** | 59 | 79 | 21 | 2154 |
| **2** | 91 | 108 | 18 | 2112 |
| **3** | 136 | 157 | 22 | 1807 |
| **4** | 181 | 197 | 17 | 1137 |
| **5** | 213 | 235 | 23 | 1451 |
| **6** | 260 | 277 | 18 | 1982 |

(f)


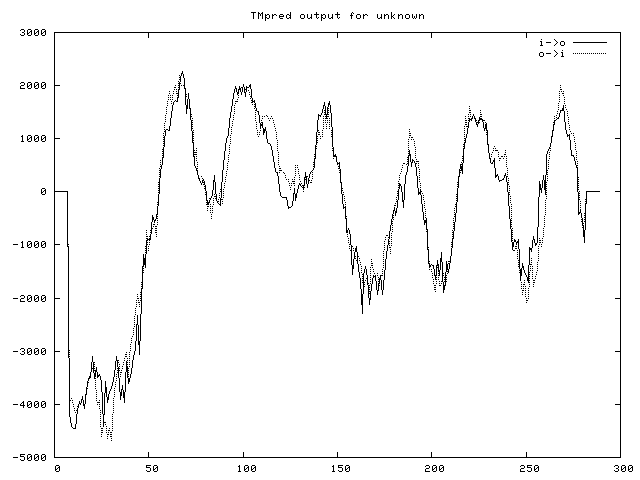


| **Transmembrane helice** | **from** | **to** | **length** | **score** |
| --- | --- | --- | --- | --- |
| **1** | 59 | 79 | 21 | 2271 |
| **2** | 91 | 108 | 18 | 1970 |
| **3** | 136 | 157 | 22 | 1692 |
| **4** | 181 | 197 | 17 | 1175 |
| **5** | 213 | 235 | 23 | 1451 |
| **6** | 260 | 277 | 18 | 1999 |

(g)


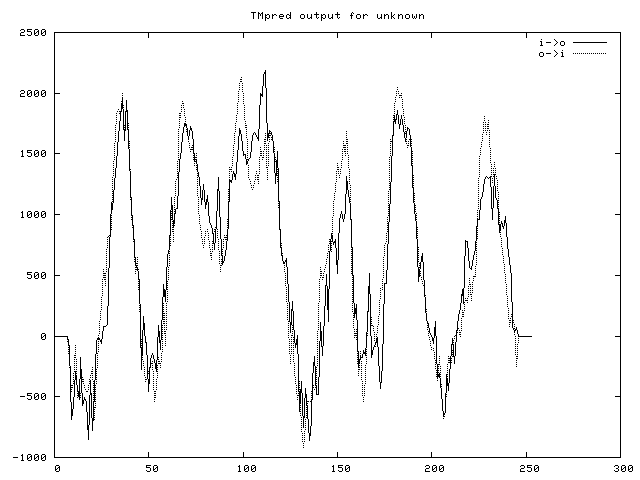


| **Transmembrane helice** | **from** | **to** | **length** | **score** |
| --- | --- | --- | --- | --- |
| **1** | 29 | 47 | 19 | 1987 |
| **2** | 60 | 79 | 20 | 1939 |
| **3** | 102 | 123 | 22 | 2186 |
| **4** | 146 | 166 | 21 | 1686 |
| **5** | 172 | 194 | 23 | 1686 |
| **6** | 219 | 237 | 19 | 1808 |

(h)


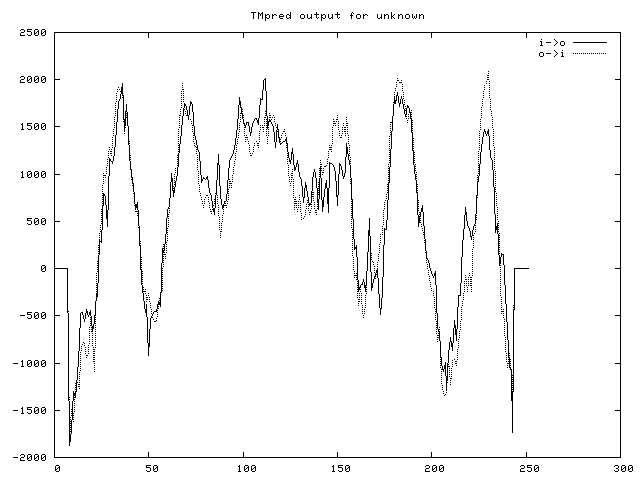


| **Transmembrane helice** | **from** | **to** | **length** | **score** |
| --- | --- | --- | --- | --- |
| **1** | 29 | 47 | 19 | 1965 |
| **2** | 60 | 77 | 18 | 1973 |
| **3** | 105 | 123 | 19 | 2010 |
| **4** | 134 | 166 | 23 | 1622 |
| **5** | 171 | 194 | 24 | 1860 |
| **6** | 219 | 241 | 23 | 2089 |


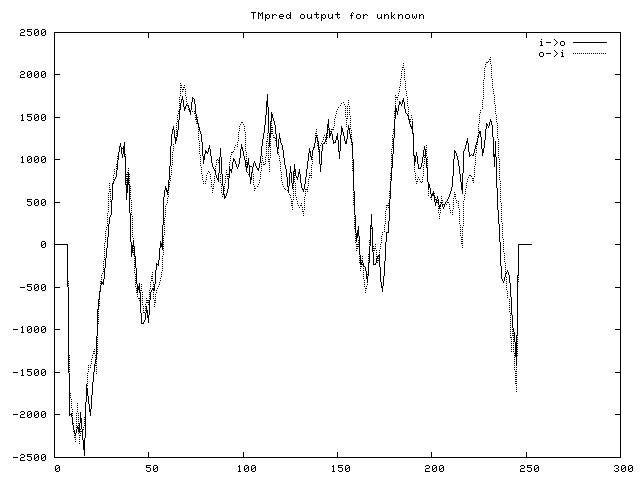
(i)

| **Transmembrane helice** | **from** | **to** | **length** | **score** |
| --- | --- | --- | --- | --- |
| **1** | 25 | 46 | 22 | 1208 |
| **2** | 59 | 78 | 20 | 1913 |
| **3** | 106 | 124 | 19 | 1768 |
| **4** | 147 | 167 | 21 | 1717 |
| **5** | 177 | 196 | 20 | 1723 |
| **6** | 220 | 242 | 23 | 2205 |

(2)

(a)


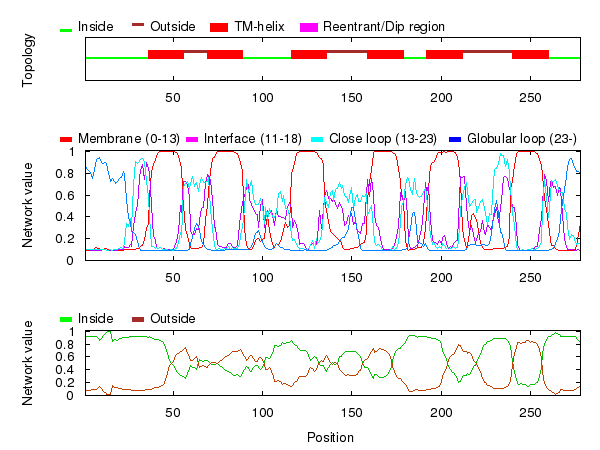


(b)


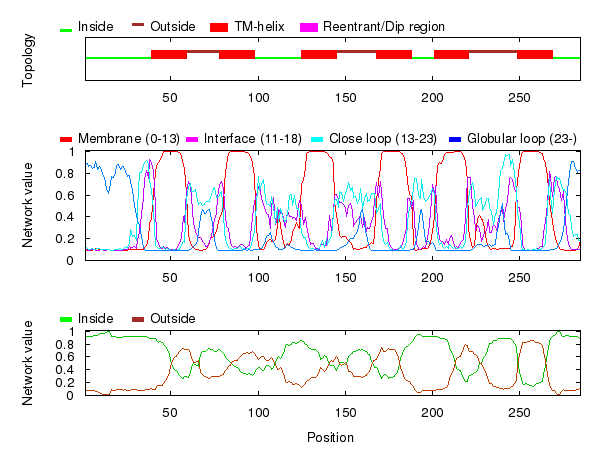


(c)


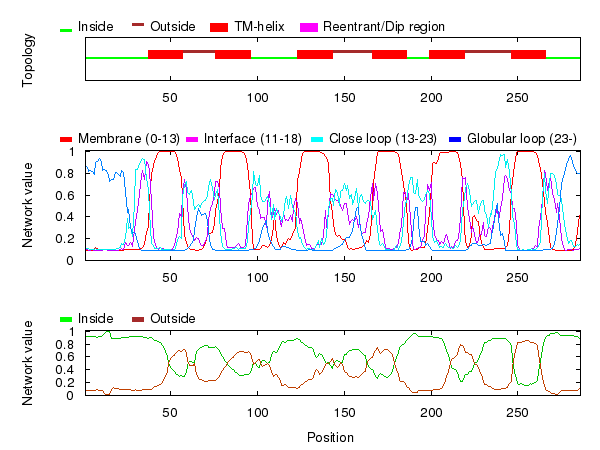


(d)


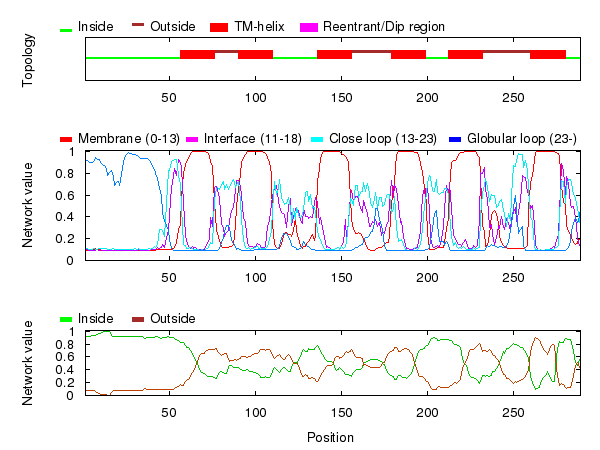


(e)


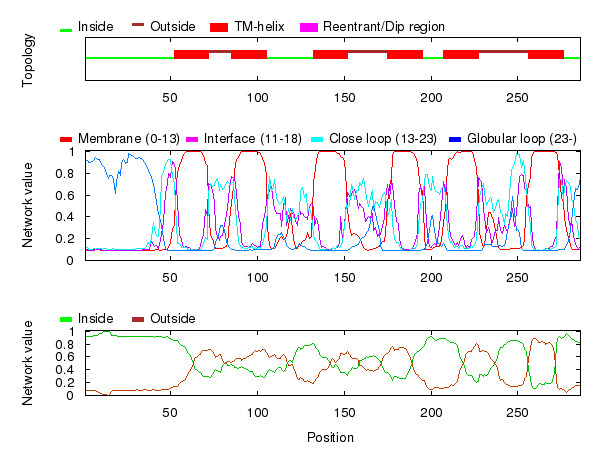


(f)


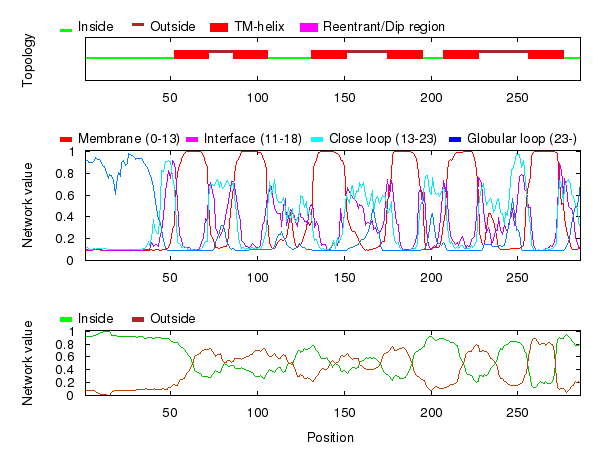


(g)


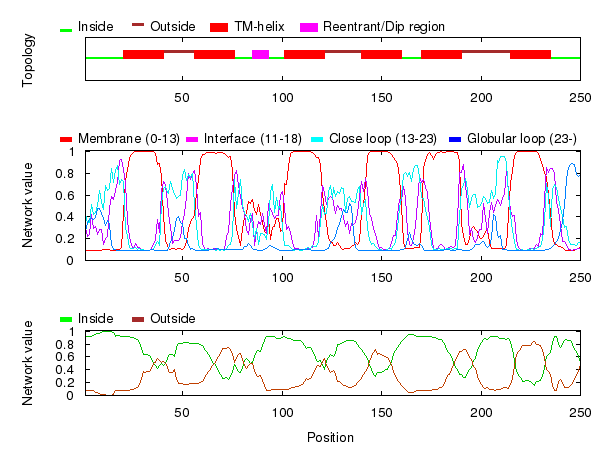


(h)


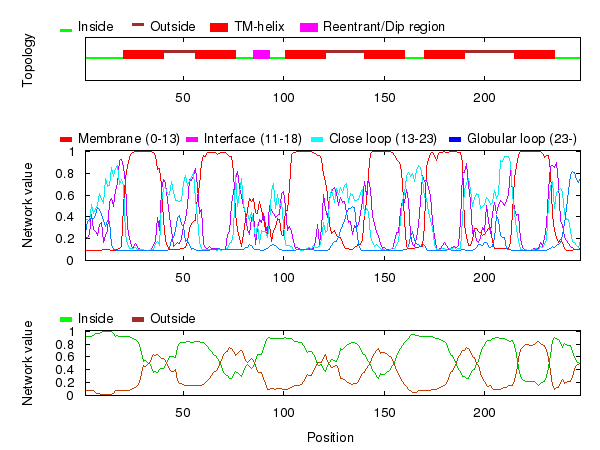


(i)


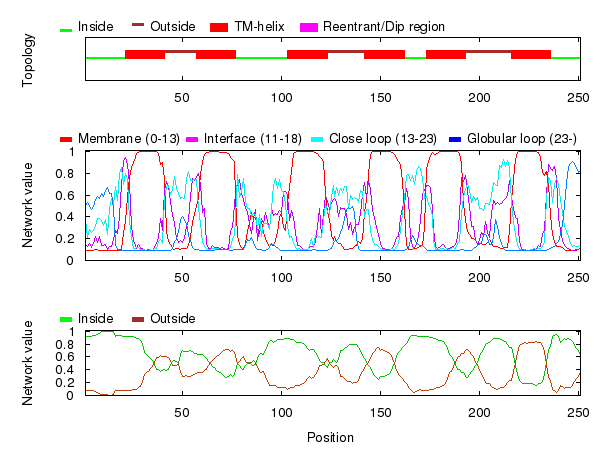

Supplement: Figure S3 — Oak AQP results from the TMpred (1) and OCTOPUS (2) servers. PIP2;1 (a), PIP2;2 (b), PIP2;3 (c), PIP1;1 (d), PIP1;2 (e), PIP1;3 (f), TIP2;1 (g), TIP2;2 (h) and TIP1 (i). Helical, membrane-spanning present peaks corresponding with the six major transmembrane domains (indicated by red lines) were predicted using TMpred. Details regarding the length and position of the transmembrane regions of the amino acid sequence are provided in the accompanying table. The presence of six transmembrane helices, marked in red, was confirmed from topology predicted using OCTOPUS and is shown in the upper schematic of the AQP topology. The green and brown loops are predicted to be located in the cytosolic and extracellular parts, respectively. The two additional red peaks in the middle graph correspond with minor helices, which were found for all oak AQPs (denoted by black arrows). (DOC) [file pone.0051838.s003.doc]
