# Supplementary material for: Identification and Expression of Nine Oak Aquaporin Genes in the Primary Root Axis of Two Oak Species, Quercus petraea and Quercus robur
Source: PLoS One. 2012 Dec 17;7(12):e51838. doi: 10.1371/journal.pone.0051838 (PMC3524086; doi:10.1371/journal.pone.0051838)
Supplement: Table S1 — The list of mRNA sequences identified from SSH libraries (a) and details of their use in RACE experiments (b). (DOC) [file pone.0051838.s004.doc]

**Table S1**

**a)**

| Gen Bank acession number | EST name | Organism | Predicted aquaporin family | Sequence use |
| --- | --- | --- | --- | --- |
| gi|166243656 | CU639735 | *Q. petraea* | PIP1 | Analysed sequence for RACE-PCR |
| gi|166243992 | CU639847 | *Q. petraea* | PIP2 | Analysed sequence for RACE-PCR |
| gi|167812277 | CU657344 | *Q. petraea* | PIP2 | Analysed sequence for RACE-PCR |
| gi|167811833 | CU656364 | *Q. petraea* | TIP2 | Analysed sequence for RACE-PCR |
| gi|167812063 | CU656415 | *Q. petraea* | PIP1 | Analysed sequence for RACE-PCR |
| gi|167812090 | CU656428 | *Q. petraea* | PIP1 | Similar to CU656415 Not considered for RACE-PCR |
| gi|167812870 | CU656596 | *Q. petraea* | PIP1 | Analysed sequence for RACE-PCR |
| gi|167812591 | CU656541 | *Q. petraea* | TIP1 | Analysed sequence for RACE-PCR |
| gi|166244330 | CU640067 | *Q. petraea* | TIP2 | Analysed sequence for RACE-PCR |
| gi|167812291 | CU657358 | *Q. petraea* | PIP1 | Analysed sequence for RACE-PCR |
| gi|167811903 | CU656755 | *Q. robur* | PIP2 | Analysed sequence for RACE-PCR |
| gi|167811876 | CU656728 | *Q. robur* | TIP1 | Analysed sequence for RACE-PCR |
| gi|167812818 | CU656237 | *Q. robur* | PIP1 | Analysed sequence for RACE-PCR |
| gi|167812478 | CU656131 | *Q. robur* | TIP2 | Analysed sequence for RACE-PCR |
| gi|166243615 | CU639694 | *Q. robur* | PIP1 | Analysed sequence for RACE-PCR |
| gi|166243831 | CU640246 | *Q. robur* | TIP2 | Analysed sequence for RACE-PCR |
| gi|167811920 | CU656772 | *Q. robur* | PIP2 | Similar to CU639847 Not considered for RACE-PCR |
| gi|167811902 | CU656754 | *Q. robur* | PIP2 | Similar to CU656755 Not considered for RACE-PCR |
| gi|167812185 | CU656915 | *Q. robur* | PIP1 | Similar to CU656415 Not considered for RACE-PCR |
| gi|167812191 | CU656921 | *Q.robur* | TIP1 | Similar to CU656541 Not considered for RACE-PCR |
| gi|166243851 | CU640266 | *Q.robur* | TIP2 | Similar to CU640067 Not considered for RACE-PCR |

**b)**

| EST name | Primer code | DNA sequence | Use |
| --- | --- | --- | --- |
| CU639735 | 735Sens | 5'-ACTTTTGGGCTGTTGTTGGCAAGGAAG-3' | 3' RACE |
| CU639735 | 735Antisens | 5'-TTACCACAGCAGCACCACAGATAGCTC-3' | 5' RACE |
| CU639847 | 847Sens | 5'-GAATAGCTTGGGCCTTTGGTGGCATG-3' | 3' RACE |
| CU639847 | 847Antisens | 5'-CCTAGCCAGGAATAGCCCGAATGTCAC-3' | 5' RACE |
| CU657344 | 344Sens | 5'-GTCTACCGCACTGCTGGAATCTCTGGT-3' | 3' RACE |
| CU657344 | 344Antisens | 5'-AGCCACAAGGTTAGCACCACCACC-3' | 5' RACE |
| CU656364 | 364Sens | 5'-TGGCCAGGATTGCCTCCACCACCA-3' | 3' RACE |
| CU656415 | 415Antisens | 5'-AAGGAAGGGCTCTGATCACAACCTGG-3' | 5' RACE |
| CU656596 | 596Antisens | 5'-AGCTGCAAGTGCTGCCCCAATGAA-3' | 5' RACE |
| CU656541 | 541Sens | 5'-TGGTGAAGGCTCTGGCATGGCT-3' | 3' RACE |
| CU640067 | 067Antisens | 5'-TCGACCCACCACTAAATGGACCAGCA-3' | 5' RACE |
| CU657358 | 358Sens | 5'-CCAGAGAGCAAGCCTATAGGGACAGC-3' | 3' RACE |
| CU657358 | 358Antisens | 5'-AGCGATCCCTTGAATTCCCACAGTTGA-3' | 5' RACE |
| CU656755 | 755Antisens | 5'-TGGTGGTAGAAGGCAGCAATGGCA-3' | 5' RACE |
| CU656728 | 728Sens | 5'-CCAATAGCGATCGGTTTCATCGTGGGA-3' | 3' RACE |
| CU656728 | 728Antisens | 5'-CCAGTAAACCCAGTTGGTTCGCCCA-3' | 5' RACE |
| CU656237 | 237Sens | 5'-TGTGACCTTTGGGCTCCTTTTGGCA-3' | 3' RACE |
| CU656237 | 237Antisens | 5'-TTCACAACGTTGGCTCCACCACCA-3' | 5' RACE |
| CU656131 | 131Antisens | 5'-ACAACAGCTGGGCCAAACGAACGA-3' | 5' RACE |
| CU639694 | 694Sens | 5'-AGCCCATAGGGACATCAGCACAGACA-3' | 3' RACE |
| CU640246 | 246Sens | 5'-TGGCACTTGGTGGCCAAATCACAGT-3' | 3' RACE |
|  | UPM | 5'-CTAATACGACTCACTATAGGGCAAGCAGTGGTATCAACGCAGAGT-3' | Universal primer Mix |
