# Supplementary material for: Identification and Expression of Nine Oak Aquaporin Genes in the Primary Root Axis of Two Oak Species, Quercus petraea and Quercus robur
Source: PLoS One. 2012 Dec 17;7(12):e51838. doi: 10.1371/journal.pone.0051838 (PMC3524086; doi:10.1371/journal.pone.0051838)
Supplement: Table S2 — Details of the real-time PCR procedure, including a list of the primers (a) and PCR conditions (b) used in the experiments. (DOC) [file pone.0051838.s005.doc]

**Table S2**

1. **Primer sequences used for real-time PCR analysis and condition of amplification**

TableS1a: List of primer sequences used for real-time PCR analysis. Primers pairs were designed using Beacon Designer Software and Primer 3 software (<http://biotools.umassmed.edu/bioapps/primer3_www.cgi>).

| **Target gene name** | Forward primer | Reverse primer |
| --- | --- | --- |
| PIP2;1 | 5’- CCGTAGCAACCCCACCAAC -3’ | 5’- AGTCATCATCATCCACATCCTCTC -3’ |
| PIP2;2 | 5’- AAGATGGAGATGGGTGTTTCA -3’ | 5’- TGACTATTGAGGATTGCTAAAGGG -3’ |
| PIP2;3 | 5’- TGATTCCTTCTTCGTGGTTC -3’ | 5’- AAGGAAATCAAGAGAGCCAAA -3’ |
| PIP1;1 | 5’- TCAGAGCCCTTCCTTTCA -3’ | 5’- GAACCAACTCCGCTTGAT -3’ |
| PIP1;2 | 5’- TCTTCTACTCTTTTCTTTGA -3’ | 5’- ATACAGCATAATTTACACAC -3’ |
| PIP1;3 | 5’-ATTCCTCTTGCATTTTATCGTT -3’ | 5’-ACACATCAATTCCAATCTCAACAC -3’ |
| TIP1 | 5’- CACTACAGACTACTGAGAATTGGA -3’ | 5’- AAACACAAAGAACAACCCTCC -3’ |
| TIP2;1 | 5’- CTCAAGACTATGCCTAAACAGAGT -3’ | 5’- ACAACCCAAATCACAGGAAGA -3’ |
| TIP2;2 | 5’- ACCTCTGTCCAATGAGTATT -3’ | 5’- GCTTCTCCTCTCCTTTATTAC -3’ |
| Actin | 5’- ACGAGCTTCCTAAATGGAGAC -3’ | 5’- CCAGCAAGTATGCCTCAAAA -3’ |
| Alpha-tubulin | 5’- GCTGAAGGAGAAGATGATGAAG -3’ | 5’- GGCAGAACACTACACCAACA -3’ |
| Cyclophylin | 5’- TACAACCCATCTATCAAA -3’ | 5’- CATTACCATCCAACCTAC -3’ |
| EF 1 alpha | 5’- GCTGTGGTGGAAACTCTGT -3’ | 5’- TCAATAATCAACTACCCGACTCAT -3’ |
| Membrane H+ ATPase | 5’- AGAGCTAAGTTAGTCTTCCTT -3’ | 5’- GGTTTCGTTCGTCATCATT -3’ |
| Polyubiquitin | 5’- CATAGGAAACCAGTTGAGG -3’ | 5’- ACAAGTTTACTGCCACATT -3’ |

**b) Details about PCR conditions for each aquaporin gene and number of primer sets tested during the optimization.**

| **Target gene name** | Practical  Hybridization temperature | Adjusted Mg Cl_2_ concentration | Number of primer set tested |
| --- | --- | --- | --- |
| PIP2;1 | 60 |  | 1 |
| PIP2;2 | 57 |  | 1 |
| PIP2;3 | 58 | Yes | 2 |
| PIP1;1 | 57 |  | 1 |
| PIP1;2 | 52 | Yes | 2 |
| PIP1;3 | 55 |  | 2 |
| TIP1 | 60 |  | 2 |
| TIP2;1 | 58 |  | 1 |
| TIP2;2 | 60 |  | 1 |
| Actin | 52 |  | 3 |
| Alpha-tubulin | 55 |  | 1 |
| Cyclophylin | 52 |  | 2 |
| EF 1 alpha | 57 |  | 1 |
| Membrane H+ ATPase | 55 | Yes | 2 |
| Polyubiquitin | 57 |  | 1 |
| **Beta-tubulin (not experimentally valited)** | na | na | 3 |

Adjustement of MgCl_2_concentration was performed increasing the concentration by step of 0.5 mM according to Roche Applied Science Customer Support Center (contact: Dr N. Viebig). An increase of 1 mM was validated for amplification of PIP2;3, PIP1;2 and Membrane H^+^ ATPase genes.
