# Supplementary material for: Identification and Expression of Nine Oak Aquaporin Genes in the Primary Root Axis of Two Oak Species, Quercus petraea and Quercus robur
Source: PLoS One. 2012 Dec 17;7(12):e51838. doi: 10.1371/journal.pone.0051838 (PMC3524086; doi:10.1371/journal.pone.0051838)
Supplement: Table S3 — List of sequences used for phylogenetic analysis. (DOC) [file pone.0051838.s006.doc]

Table S3

| **Protein ID UNIPROT** | **NCBI WGS contig accession number** | **JGI accession number** | **AGI code** | **Organism** | **Abbreviation** |
| --- | --- | --- | --- | --- | --- |
| P61837 |  |  | At3g61430 | *Arabidopsis thaliana* | *At*PIP1 :1 |
| Q06611 |  |  | At2g45960 | *Arabidopsis thaliana* | *At*PIP1 :2 |
| Q08733 |  |  | At1g01620 | *Arabidopsis thaliana* | *At*PIP1 :3 |
| Q39196 |  |  | At4g00430 | *Arabidopsis thaliana* | *At*PIP1 :4 |
| Q8LAA6 |  |  | At4g23400 | *Arabidopsis thaliana* | *At*PIP1 :5 |
| P43286 |  |  | At3g53420 | *Arabidopsis thaliana* | *At*PIP2 :1 |
| P43287 |  |  | At2g37170 | *Arabidopsis thaliana* | *At*PIP2 :2 |
| P30302 |  |  | At2g37180 | *Arabidopsis thaliana* | *At*PIP2 :3 |
| Q9FF53 |  |  | At5g60660 | *Arabidopsis thaliana* | *At*PIP2 :4 |
| Q9SV31 |  |  | At3g54820 | *Arabidopsis thaliana* | *At*PIP2 :5 |
| Q9ZV07 |  |  | At2g39010 | *Arabidopsis thaliana* | *At*PIP2 :6 |
| P93004 |  |  | At4g35100 | *Arabidopsis thaliana* | *At*PIP2 :7 |
| Q9ZVX8 |  |  | At2g16850 | *Arabidopsis thaliana* | *At*PIP2 :8 |
| P25818 |  |  | At2g36830 | *Arabidopsis thaliana* | *At*TIP1 :1 |
| Q41963 |  |  | At3g26520 | *Arabidopsis thaliana* | *At*TIP1 :2 |
| O82598 |  |  | At4g01470 | *Arabidopsis thaliana* | *At*TIP1 :3 |
| Q41951 |  |  | At3g16240 | *Arabidopsis thaliana* | *At*TIP2 :1 |
| Q41975 |  |  | At4g17340 | *Arabidopsis thaliana* | *At*TIP2 :2 |
| Q9FGL2 |  |  | At5g47450 | *Arabidopsis thaliana* | *At*TIP2 :3 |
|  | AARH01004386 | 724520 |  | *Populus trichocarpa* | *Pt*PIP1 :1 |
|  | AARH01003541 | 656216 |  | *Populus trichocarpa* | *Pt*PIP1 :2 |
|  | AARH01001832 | 711735 |  | *Populus trichocarpa* | *Pt*PIP1 :3 |
|  | AARH01003029 | 831918 |  | *Populus trichocarpa* | *Pt*PIP1 :4 |
|  | AARH01006875 | 835561 |  | *Populus trichocarpa* | *Pt*PIP1 :5 |
|  | AARH01003794 | 821084 |  | *Populus trichocarpa* | *Pt*PIP2 :1 |
|  | AARH01002541 | 648808 |  | *Populus trichocarpa* | *Pt*PIP2 :2 |
|  | AARH01004412 | 567607 |  | *Populus trichocarpa* | *Pt*PIP2 :3 |
|  | AARH01003539 | 563742 |  | *Populus trichocarpa* | *Pt*PIP2 :4 |
|  | AARH01008299 | 826419 |  | *Populus trichocarpa* | *Pt*PIP2 :5 |
|  | AARH01008299 | N/A |  | *Populus trichocarpa* | *Pt*PIP2 :6 |
|  | AARH01006751 | 735495 |  | *Populus trichocarpa* | *Pt*PIP2 :7 |
|  | AARH01003912 | 821627 |  | *Populus trichocarpa* | *Pt*PIP2 :8 |
|  | AARH01008738 | 796664 |  | *Populus trichocarpa* | *Pt*PIP2 :10 |
|  | AARH01000429 | 549212 |  | *Populus trichocarpa* | *Pt*TIP1 :1 |
|  | AARH01003864 | 833283 |  | *Populus trichocarpa* | *Pt*TIP1 :2 |
|  | AARH01004405 | 822504 |  | *Populus trichocarpa* | *Pt*TIP1 :3 |
|  | AARH01003540 | 656044 |  | *Populus trichocarpa* | *Pt*TIP1 :4 |
|  | AARH01006799 | 667870 |  | *Populus trichocarpa* | *Pt*TIP1 :5 |
|  | AARH01008323 | 589502 |  | *Populus trichocarpa* | *Pt*TIP1 :6 |
|  | AARH01003929 | 558321 |  | *Populus trichocarpa* | *Pt*TIP1 :7 |
|  | AARH01010693 | 828458 |  | *Populus trichocarpa* | *Pt*TIP1 :8 |
|  | AARH01000349 | 548890 |  | *Populus trichocarpa* | *Pt*TIP2 :1 |
|  | AARH01001731 | 645978 |  | *Populus trichocarpa* | *Pt*TIP2 :2 |
