# Supplementary material for: Identification and Expression of Nine Oak Aquaporin Genes in the Primary Root Axis of Two Oak Species, Quercus petraea and Quercus robur
Source: PLoS One. 2012 Dec 17;7(12):e51838. doi: 10.1371/journal.pone.0051838 (PMC3524086; doi:10.1371/journal.pone.0051838)
Supplement: Table S6 — Protein sequence homology between oak AQPs based on isolated cDNAs. Sequence homology was determined using ClustalW (http://www.ebi.ac.uk/Tools/msa/clustalw2/). (DOC) [file pone.0051838.s009.doc]

**Table S5**

| Aquaporin gene | PIP2;1 | PIP2;2 | PIP2;3 | PIP1;1 | PIP1;2 | PIP1;3 | TIP2;1 | TIP2;2 | TIP1 |
| --- | --- | --- | --- | --- | --- | --- | --- | --- | --- |
| PIP2;1 | 100 |  |  |  |  |  |  |  |  |
| PIP2;2 | 83 | 100 |  |  |  |  |  |  |  |
| PIP2;3 | 76 | 82 | 100 |  |  |  |  |  |  |
| PIP1;1 | 67 | 67 | 66 | 100 |  |  |  |  |  |
| PIP1;2 | 68 | 67 | 66 | 86 | 100 |  |  |  |  |
| PIP1;3 | 67 | 67 | 65 | 87 | 91 | 100 |  |  |  |
| TIP2;1 | 31 | 30 | 30 | 27 | 28 | 30 | 100 |  |  |
| TIP2;2 | 30 | 32 | 31 | 28 | 29 | 31 | 76 | 100 |  |
| TIP1 | 27 | 29 | 29 | 25 | 25 | 25 | 60 | 62 | 100 |
